# Charting Alzheimer’s Disease and Dementia: Epidemiological Insights, Risk Factors and Prevention Pathways

**DOI:** 10.3390/jcm13144100

**Published:** 2024-07-13

**Authors:** Israel Contador, Bárbara Buch-Vicente, Teodoro del Ser, Sara Llamas-Velasco, Alberto Villarejo-Galende, Julián Benito-León, Félix Bermejo-Pareja

**Affiliations:** 1 Department of Basic Psychology, Psychobiology, and Methodology of Behavioral Sciences, Faculty of Psychology, University of Salamanca, 37005 Salamanca, Spain; 2 Aging Research Center, Department of Neurobiology, Care Sciences and Society, Karolinska Institutet and Stockholm University, 17117 Stockholm, Sweden; 3 Alzheimer Centre Reina Sofia—CIEN Foundation, Institute of Health Carlos III, 28031 Madrid, Spain; tdelser@fundacioncien.es; 4 Instituto de Investigación Sanitaria Hospital 12 de Octubre (imas12), 28041 Madrid, Spain; sara.llamas@salud.madrid.org (S.L.-V.); alberto.villarejo@salud.madrid.org (A.V.-G.); julian.benito@salud.madrid.org (J.B.-L.); 5 Department of Neurology, University Hospital 12 de Octubre, 28041 Madrid, Spain; 6 Centro de Investigación Biomédica en Red Sobre Enfermedades Neurodegenerativas (CIBERNED), 28029 Madrid, Spain; 7 Department of Medicine, Faculty of Medicine, Complutense University, 28040 Madrid, Spain

**Keywords:** Alzheimer’s disease, prevention, risk factors, public health, epidemiology

## Abstract

Alzheimer’s disease (AD), the most common cause of dementia, is a complex and multifactorial condition without cure at present. The latest treatments, based on anti-amyloid monoclonal antibodies, have only a modest effect in reducing the progression of cognitive decline in AD, whereas the possibility of preventing AD has become a crucial area of research. In fact, recent studies have observed a decrease in dementia incidence in developed regions such as the US and Europe. However, these trends have not been mirrored in non-Western countries (Japan or China), and the contributing factors of this reduction remain unclear. The Lancet Commission has delineated a constrained classification of 12 risk factors across different life stages. Nevertheless, the scientific literature has pointed to over 200 factors—including sociodemographic, medical, psychological, and sociocultural conditions—related to the development of dementia/AD. This narrative review aims to synthesize the risk/protective factors of dementia/AD. Essentially, we found that risk/protective factors vary between individuals and populations, complicating the creation of a unified prevention strategy. Moreover, dementia/AD explanatory mechanisms involve a diverse array of genetic and environmental factors that interact from the early stages of life. In the future, studies across different population-based cohorts are essential to validate risk/protective factors of dementia. This evidence would help develop public health policies to decrease the incidence of dementia.

## 1. Introduction

Dementia is one of the leading causes of dependency and disability worldwide [1]. It is estimated that more than 50 million people suffer from this condition, with Alzheimer’s disease (AD) being the primary dementia subtype (60–70%) [2]. Due to the aging of the global population, it is expected that the prevalence of dementia will rise to 131.5 million cases by 2050, especially in low- and middle-income countries [3]. The World Health Organization (WHO) estimates dementia cost around USD $818 billion in 2015, equivalent to 1.1% of global gross domestic product, ranging from 0.2%—low- and middle-income countries—to 1.4%—high-income countries [4,5]. Therefore, dementia is considered a global public health priority.

Currently, it is well-known that AD is a chronic brain disorder with a long silent period of decades (preclinical phase) before its clinical onset [6,7,8]. This fact, linked to the failure of pharmacological AD therapies, has boosted research on dementia/AD prevention [9]. Thus, recent studies have shown that the age-specific incidence of dementia is unexpectedly decreasing in some countries [3,10,11,12,13], but explanatory factors remain undetermined, and further evidence is needed to resolve this enigma [14,15,16,17]. During the last few years, there has been growing interest in preventive approaches based on controlling dementia/AD risk factors [18,19]. A scientific report has recently identified 12 modifiable dementia risk factors (i.e., low education, arterial hypertension, hearing loss, smoking, obesity, depression, physical inactivity, diabetes mellitus, low social contact, excessive alcohol consumption, head injury, and air pollution), which collectively account for almost 40% of the worldwide burden of dementia [15]. Accordingly, the WHO has already issued guidelines on risk reduction of cognitive decline and dementia [5]. At the same time, various medical societies have proposed a second generation of memory clinics (Brain Health Services) aimed at developing evidence-based counseling for dementia prevention [20,21,22,23]. These efforts have the potential to significantly reduce socioeconomic dementia costs; For instance, the reduction of the incidence of AD by 20% by 2050 is estimated to save Europe up to EUR 33 billion [24].

The main objective of this review is to outline the current state of evidence concerning the modifiable factors associated with dementia/AD. Specifically, we examine epidemiological trends across different cohorts, risk and protective modifiable factors, and general strategies to delay dementia onset.

## 2. Methods

This research is a critical narrative review compiling several miscellaneous searches in PubMed using the following Medical Subjects Headings (MeSH): “Dementia/AD risk factors”, “dementia/AD epidemiology”, “dementia/AD meta-analysis”, and “dementia/AD review”. The leading search was conducted between May and December 2023, and various combinations of Boolean operators were used, along with other search strategies (i.e., backward reference searching), to enhance the quality of the search. English was the primary language for the selection, although high-quality scientific papers in other languages were also reviewed. All authors, who are experts in the field, approved the final information.

## 3. Is the Incidence of Dementia on the Rise or Declining?

Manton et al. [25] provided initial evidence of declining prevalence (from 5.7% to 2.9%) of dementia in the elderly population of the United States (national register) between 1982 and 1999. This reduction was significant in mixed and vascular dementias, but not in AD. Subsequently, Rocca et al. (2011) [26] conducted a study across various communities in the USA, revealing a decline in the incidence of dementia and cognitive impairment over 10–20 years. Since then, new data on dementia prevalence and incidence have emerged, mainly from population-based cohorts. Table 1 shows the decreasing incidence of dementia among older adults over the past two decades in developed Western countries, a consistent trend in scientific reviews and worldwide reports, including in non-Western countries [13,27,28]. However, other developed non-Western nations such as Japan [29], Korea [30], and China [31] have shown an increased prevalence rates of dementia. In addition, rigorous surveys on dementia trends are lacking in Africa, with the exception of Nigeria, and Latin America [32]. The following epigraphs describe the main RF and PF of dementia.

## 4. Risk Factors

The term “risk factors” (RFs) is a concept widely used in epidemiology that has gained prominence in the field of cardiovascular diseases (see Framingham Heart Study, 1948) [3]. This landmark of cardiovascular research, with an exceptionally long-term follow-up, raised awareness about the identification of RFs and their importance for preventing diseases [62]. Data from this pioneering study and other comparable cohorts marked the beginning of a paradigm shift—the era of prevention, which highlights the importance of lifestyle as the cornerstone of health. Consequently, terms such as “healthy aging” or “successful aging” have been more frequent in scientific publications since the 1980s. Even the World Health Organization (WHO), at the First International Conference on Health Promotion (Ottawa, 1986), emphasized that individual responsibility and health promotion are essential for healthy aging. Since then, significant progress has been made in the field of disease prevention, including dementia/AD.

### 4.1. Genetic Risk Factors

The heritability of dementia/AD is still poorly understood [63]. Basically, most of the genetic analyses have been performed in clinically diagnosed AD, while studies in other dementias (mainly vascular) are quite limited [64,65].

In this context, genetic forms of AD, which account for approximately 1% of cases, are due to monogenic mutations that lead to early dementia onset. Most of these mutations (70%) are found in the presenilin 1 gene (chromosome 14). Other known mutations occur in the presenilin 2 gene (chromosome 1) and the APP gene (chromosome 21) [66]. These AD forms exhibit dominant heritability with very high penetrance, but other early AD cases may also have recessive heritability [67]. In contrast, the heritability of sporadic AD cases (i.e., non-familial and usually with a later onset—over 65 years) has a complex polygenic pattern and interacts with diverse environmental RFs [68].

The ApoE gene is a significant susceptibility factor for all forms of AD. It has three major allelic forms—ε2, ε3, and ε4—with the ε4 allele significantly increasing the risk for both familial and sporadic AD [66]. Current research has found that over 95% of individuals (aged 65 years and older) with two copies of the ApoE ε4 gene (i.e., homozygotes) show biological characteristics of AD pathology(abnormal amyloid levels in cerebrospinal fluid) [69]. In addition, these homozygous individuals also develop the disease earlier than those with other variants of the ApoE gene. Nevertheless, the ε2 allele and the Christchurch (APOE3Ch) variant in ε3 may provide resistance to AD [70]. It is worth mentioning that heritability is context-specific, being influenced by ethnicities and ancestries [63,71]. Therefore, caution should be employed when applying genetic findings on AD to diverse populations.

Genetic studies have consistently demonstrated that sporadic AD has a significant heritability (i.e., phenotypic variance attributable to genetics). According to the most extensive twin studies, the Swedish HARMONY [72] and the Finnish twin study [64], sporadic AD has a heritability of 58–79% and 60%, respectively. Most twins share genetic and environmental RFs, with a portion of heritability attributed to the interaction between genes and environment -epigenetic changes- [73,74,75,76]. However, one of the issues in the genetics of AD is the “missing heritability” (i.e., the difference between the clinical heritability from twin studies versus the genome-wide association studies [GWAS] of single nucleotide polymorphisms) [77,78,79]. In fact, GWAS studies quantify the heritability of AD between 3% and 53% [71], which represents less than one-third of the clinical heritability. The unexplained heritability in AD, a phenomenon observed in other complex disorders (diabetes mellitus and schizophrenia), may result from the interaction between genes (i.e., epistasis), epigenetic changes, or rare causal variants of small effect [75,80].

In the last decade, our knowledge of AD heritability has been mainly based on the GWAS, which may detect many genetic polymorphisms (SNP) associated with developing AD [81]. This technique has identified up to 73 genetic loci related to AD [82], the majority established in Caucasian ancestries, whereas other loci have been described in other non-western populations such as China [83] or Africa [78]. The weighted sum of the estimated effects of these multiple genetic variants associated with AD can be calculated as an individual polygenic risk score (PRS) [84]. The establishment of a PRS is a very complex genetic work, mainly in dementia/AD, but it may have eventual practical application for prognosis or therapy [17,85]. Extensive databases of dementia patients (mainly AD) and controls are being created in the USA and Europe. The UK Biobank, comprising nearly 200,000 individuals genetically tested [86,87], and the Health and Retirement Study in the USA (>10,000 individuals), are some examples [88]. In this context, some studies have developed specific PRS for dementia and AD [89,90,91], but they are currently restricted to research [63].

### 4.2. Early Risk Factors

Barker et al. [92] described the relationship between low birthweight and early mortality by myocardial infarction. This finding posited the possibility that infant undernutrition was associated with developing chronic disorders in later life periods. This evidence is consistent with the controversial hypothesis of developmental origins of health and disease (DOHaD) [93,94]. Borenstein et al. [95] reviewed this issue for dementia and AD, concluding that the risk of AD is likely not determined by a single period but rather a complex interplay between genetic and environmental exposures throughout all the life course. It has been claimed that infant undernutrition [96,97,98] is an early RF for cognitive decline and dementia, although its impact can be mediated by epigenetic mechanisms [99]. In addition, many studies, including birth registries, cohorts, and systematic reviews, have demonstrated that early life adversities (i.e., toxicities, low education, birth problems, food deficiency, body growth, brain development, and poor learning abilities) are related to mental disorders, cognitive decline, and dementia/AD [100,101,102,103,104]. Other longitudinal studies also emphasize the impact of socioeconomic-related factors—low parental education, poverty, famine, and/or maternal drug consumption [105,106,107,108,109,110]— on cognitive functioning and dementia/AD risk at later life periods. Experimental animal studies confirm the importance of early life events on cognitive impairment and AD [111,112], which may act through complex and synergistic biological mechanisms [111,113]. Despite these facts, early-life RFs have been ignored in some RFs taxonomies of dementia/AD [15]. 

### 4.3. Preventable vs. Non-Preventable Risk Factors

There is a lack of consensus on the extent to which dementia is preventable, and explanatory factors remain unclear [15,112,114]. The initial efforts of Henderson [115] and the EURODEM group (1991) [116] identified over 200 RFs based on case studies. Basically, dementia RFs have been classified into modifiable and non-modifiable categories. On the one hand, non-modifiable factors refer to those inherent to the individual, which cannot be altered through any specific actions, thereby limiting their direct prevention (e.g., genetic factors, gender, age). On the other hand, modifiable factors are those that can be influenced by individual’s behaviors, making them open to intervention and preventive strategies. Thus, Framingham’s study observed that dementia incidence has decreased within the last 30 years, making education a critical explanatory factor [3]. Other investigations are focused on health behaviors (e.g., healthy diet or cardiovascular risk management) that may reduce the risk of cognitive decline and dementia [117,118,119,120,121,122]. In addition, recent reviews underscore the influence of good living conditions and healthcare on dementia onset [13]. It is noteworthy that research in the field is typically based on epidemiological studies, making it difficult to avoid reverse causality or the exhaustive control of confounders [123].

**Figure 1:**
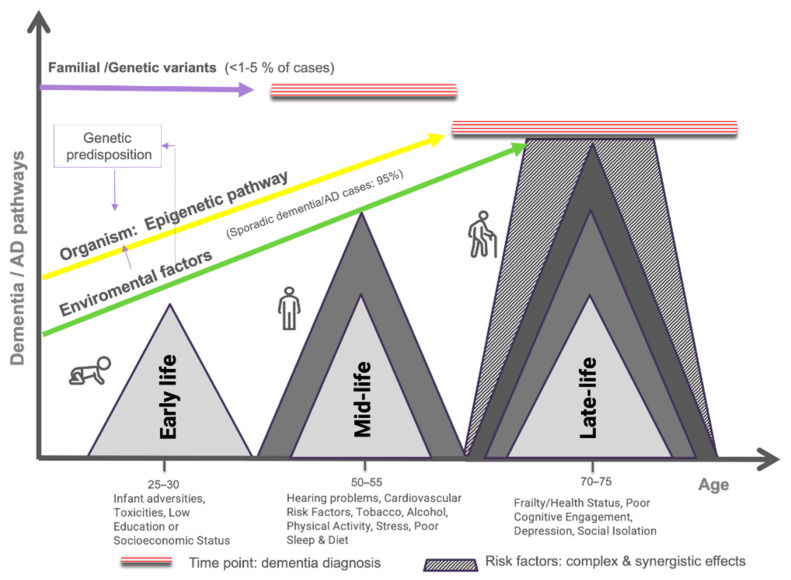
Dementia pathways and main modifiable risk factors across lifespan. The familial dementia category represents a minimal percentage of cases directly explained by autosomal gene mutations. In contrast, modifiable factors may exert their effects on different biological systems of the organism (genes, immunological response, and brain connectivity) at different lifespan periods, raising the likelihood of sporadic dementia/AD. In addition to their weighted specific effects, risk factors may interact in a synergistic and complex way with different lifestyle habits (e.g., diet, sleep), population factors (e.g., air pollution, health system), or unknown conditions converging at late life (represented by the dotted area). Modifiable risk factors may interact with genes (epigenetic pathway is shown in yellow), boosting the probability of AD/dementia compared to those sporadic cases without these traits (green line). It should be noted that dementia onset can vary significantly between individuals at different paths, but it is represented in a simplified visual manner for the readers (see red dotted area).

Recently, the Lancet Commission, a group of specialized research experts addressing global concerns, conducted a review of modifiable factors that could impact dementia onset. It presented an initial report identifying nine factors [112]: low education, hypertension, hearing impairment, smoking, obesity, depression, physical inactivity, diabetes, and low social contact. Three years later, a new report of this Commission added three factors to the list [15]: excessive alcohol consumption, traumatic brain injury (TBI), and air pollution. The report of this group of experts states that these factors collectively account for approximately 40% of worldwide dementia cases [15]. This report is consistent with recent reviews, which underscores the varying impact of these modifiable factors, depending on the individual’s life period [15,124], and the importance of prevention actions based on the individual’s behavior. To summarize, Figure 1 displays different pathways to developing dementia and prominent modifiable risk factors at different life stages.

### 4.4. Risk Factors: Individual vs. Population Factors

An essential differentiation is the individual versus population-based RFs. The individual factors include genetics, early life conditions, medical issues, lifestyle, and psychosocial aspects. Environmental, socioeconomic, cultural, and public health conditions are population-based RF. Accordingly, the implementation of robust public policies and programs addressing these factors for health promotion could effectively reduce the burden of dementia. Table 2 summarizes the most important reviews and meta-analyses on dementia/AD risk factors.

### 4.5. Individual Factors

#### 4.5.1. Education and Cognitive Stimulating Activities

Education is a critical factor influencing the risk of dementia. Lower levels of education are consistently associated with a higher risk of dementia [15,154]. In fact, some authors consider education as the most relevant dementia RF [15,135,140,155]. Moreover, engagement in cognitively stimulating activities throughout life can enhance cognitive reserves and protect from dementia [156,157]. These factors may enable the brain to actively confront damage by promoting compensatory mechanisms through the activation of alternative brain networks or mental strategies that delay the clinical expression of brain damage [158,159]. The effects of education and cognitively stimulating activities on neural tissue may entail complex and diverse mechanisms such as neurogenesis [160,161,162], angiogenesis [163], synaptic density [164,165], or neural connectivity [166,167,168,169,170].

#### 4.5.2. Medical Conditions

- Cardiovascular Diseases (CVDs)

Longitudinal research, including the well-known Framingham Study, has demonstrated that arterial hypertension increases the risk of dementia/AD [171], with the midlife period being critical for its development [172,173]. A recent meta-analysis highlighted that systolic hypertension is associated with an increased risk of AD by 18% and 25% in Stages 1 and 2 of hypertension respectively [172]. Antihypertensive drugs may reduce dementia incidence and cognitive decline [57,174]; howeverthese effects are not only due to the mere decrease in blood pressure but also the preservation of brain-vessels interaction and direct action of some hypotensive drugs on neural function [175,176]. In addition, recent reviews and meta-analyses have demonstrated that total cholesterol [177], low ankle-brachial index—peripheral artery disease [178,179], body mass index—underweight and overweight in midlife [114,119,180], high homocysteine levels [105,181], low handgrip strength [182], and metabolic syndrome [183] are significantly associated with dementia, but few studies have examined AD risk combining different cardiovascular factors [184,185]. Finally, population-based studies have underlined the influence of mid-life obesity, which has been linked to vascular dementia in particular [119,186]. Significant weight changes (underweight or obesity) in later life may be associated with dementia risk, but this fact should be taken with caution because the relationship between weight change and dementia is non-linear and depends on the dementia subtype [119,187]. The interactions with comorbidities/associated illness or reverse causality effects are likely explanatory factors of this relationship.

Basically, CVDs hinder the proper brain interaction with blood vessels and the brain’s natural processes for clearing neurotoxic waste. In addition, vascular conditions are associated with neuroinflammation and cerebral perfusion alterations [188]. Moreover, VRFs such as hypertension, diabetes, and obesity, especially in midlife, accelerate brain aging (progression of vascular pathology, global, and hippocampal atrophy) and cognitive decline [189]. It is worthwhile to consider that the impact of VRFs is multifaceted and may simultaneously promote microvascular disease, structural brain changes, and AD pathology. Indeed, higher levels of VRFs have also been associated with poorer brain health across grey and white matter brain structures [190]. Lastly, VRFs have also been associated with greater amyloid-β (Aβ) and tau burden [191], but these relationships remain controversial [192].

- Diabetes mellitus

Diabetes mellitus (DM), particularly Type 2 DM [112,193], is also associated with an increased risk of dementia [194,195] and mild cognitive impairment (MCI) in worldwide studies [193,196]. The conversion rate from MCI to dementia is higher in people with diabetes, although the duration and severity of the disease may modulate this relationship [197]. Diabetes increases dementia risk by up to 35% in a vulnerable population with ApoE ε4 allele [198]. Post-mortem evidence suggests that individuals with AD and Type 2 diabetes are more likely to have both AD-type and cerebrovascular pathologies [188], although other researchers assert that diabetes may increase dementia risk through interactions with other associated biological mechanisms (e.g., inflammation, mitochondrial dysfunction) [135]. Finally, few studies have analyzed the influence of prediabetes or insulin-resistance states on dementia risk, making this association inconclusive [114,145].

- Hearing impairment

Hearing loss in midlife (age 45–65 years) has been associated with cognitive decline and dementia [15,199], especially in those individuals with ApoE ε4 [200]. Pathology affecting the ascending auditory pathway and multimodal cortex, the depletion of cognitive reserves due to an impoverished listening environment, and the abnormal auditory processing in the temporal lobe may be responsible for these associations [201]. However, the specific mechanisms underlying the association between hearing loss and cognitive decline remain undetermined [202,203,204].

- Neurological diseases

Neurological conditions may increase dementia risk, although specific pathways to different dementia subtypes are not established. 

Current evidence suggests that individuals with a history of TBI are more vulnerable to suffering dementia [15,205,206,207,208], especially Parkinson’s disease and Lewy body subtypes. However, data on AD are inconsistent [206,209]. Dysfunction of the blood–brain barrier, mitochondrial function, β-amyloid pathology, chronic neuroinflammation, tau deposition, vascular damage, and white-matter degeneration have been suggested to explain the link between TBI and neurodegeneration [200].

Stroke is linked to vascular and AD dementia subtypes [210]. AD patients may have stroke events [211]. It is known that at least 20% of patients with stroke develop dementia at 3 months [212]. A history of previous stroke is not unusual for post-stroke dementia [145], whereas around 15% of stroke patients have pre-stroke dementia [213]. Hence, there is a mutual risk relationship between dementia and stroke, which primarily share modifiable risk and protective factors [118]. It is noteworthy that vascular conditions are associated with neuroinflammation and alterations in cerebral perfusion, resulting in increased gray matter atrophy [201]. However, the association between VRFs with greater amyloid-β (Aβ) and tau burden remains controversial [159,192].

Other neurological symptoms, such as migraines or pain, are being investigated as potential RFs for cognitive impairment [214].

- Epilepsy

Various researchers have underlined the possible relations between epilepsy and AD [147,202]. Two recent meta-analyses have determined that late-onset epilepsy is a dementia RF [215]. In fact, seizures are related to all causes of dementia, including AD [216]. A recent review underlines that epilepsy and AD share pathophysiological mechanisms (e.g., hyperexcitability and excitatory–inhibitory dysregulation), leading to dysfunctions in the GABAergic and glutamatergic systems [217].

- Depression and Anxiety

Depression is a widely accepted RF for dementia/AD [15,112,114,202,218,219,220]. However, it is challenging to ascertain whether old-age depression acts as an RF or represents an initial psychological symptom of an underlying neurodegenerative process [15,140,147]. It is known that depression may impact brain circuits at midlife, inducting distinct forms of neural dysfunctions [221], although not all depression subtypes will have the same effect [134,141]. The severity and long-term maintenance of symptoms are essential to induce neurobiological changes (e.g., increased Aβ levels) linked to an increased dementia risk [222]. In addition, anxiety may be associated with dementia markers such as amyloid or tau [223,224], increasing the probability of vascular dementia and AD [225]. Finally, recent evidence indicates that stressful life events (e.g., loss of a parent, psychological stress in midlife, post-traumatic stress disorder) correlate with a higher risk of dementia [226].

- Sleep disorders

A recent meta-analysis conducted by Bubu et al. [227], including nearly 70,000 participants, estimates that individuals with sleep problems—including poor quality and short and long sleep duration—show a higher risk of developing cognitive impairment and/or AD. Other high-quality research confirms that daily hours—short and long sleep duration—of sleep may be related to cognitive decline [228,229]. Accordingly, limited sleep duration significantly predicted higher t-tau and p-tau in older adults, mainly observed in *APOE* ε4 carriers [230]. In general, sleep disturbances have been linked to different subtypes of dementia, such as vascular and AD [231,232]. Hypoxia associated with sleep disturbances is a major contributor to neurodegenerative changes [233].

- Frailty/Poor health

Poor health status and frailty are associated with increased comorbidities in older adults, including neurocognitive deficits [234,235,236] and AD [114]. Recent models indicate that cognitive frailty is driven by dysregulation across multiple cellular processes, such as genetic alterations, metabolism of nutrients and lipids, and higher levels of pro-inflammatory proteins [237]. Furthermore, frailty interacts with AD pathology, showing that individuals with a low level of AD pathology may be at a higher risk for dementia if the level of frailty is high [238].

### 4.6. Lifestyle/Environment Preventable Risk Factors

#### 4.6.1. Alcohol Consumption

A recent study that followed 40,435 subjects for 27 years revealed that frequent alcohol consumption was significantly associated with dementia [239]. This observation is corroborated by other studies, indicating that alcohol abuse disorder is linked to cognitive impairment and dementia [240]. Actually, substantial alcohol consumption, which is associated with other dementia RFs—education, tobacco smoking, and depression — has neurotoxic effects and may lead to structural and functional brain damage [241]. Furthermore, a recent meta-analysis discerned that dementia risk varies depending on the doses and types of ethanol consumed [242]. Thus, modest alcohol consumption (≤12.5 g/day) was associated with a reduced risk of dementia, with wine being a more suitable alcohol type. Apparently, dementia risk is increased when consumption exceeds 21 units (168 g) of alcohol weekly versus lighter drinking [15]. Different observational studies have found that light-to-moderate alcohol consumption is associated with a decreased risk of cognitive impairment and dementia [114,132,243,244,245]. Still, there are contradictory findings, and a direct causality of this association has not been traced [241]. Remarkably, the WHO advocates for the reduction of alcohol consumption [5], as it is considered a direct contributor to more than 200 diseases, including dementia. Likewise, the NICE guidelines [246] also recommend minimizing alcohol consumption, especially in midlife, to mitigate the risk of age-related diseases and frailty.

#### 4.6.2. Smoking

Smoking has been widely linked to numerous diseases, including dementia and AD [15,247,248]. Hence, exposure to cigarette smoking is associated with higher amyloid-β (Aβ42) levels, excessive oxidative stress, neuroinflammation, and neurodegeneration, which may increase the probability of dementia [249]. Controversially, in some cohort studies, this risk appeared to be more pronounced in individuals who were non-carriers of the ApoE ε4 genotype [248]. The authors suggest that smoking effects may be masked in ApoE ε4 individuals due to the predominant genetic risk effect.

#### 4.6.3. Dietary Patterns

It is known that unfavorable nutrition increases the risk of dementia. For instance, a poor diet based on saturated fats may lead to cardiovascular abnormalities, increasing the risk of AD by up to 39% [250]. There is also growing data indicating that gut dysbiosis can trigger metabolic diseases and the progression of low-grade systemic inflammation, and that it is involved in many of the major modifiable dementia RFs [251]. In contrast, diverse population studies have determined that adherence to particular healthy dietary patterns, such as the Mediterranean diet, can reduce the incidence of dementia [117,252]. The prevention of oxidative stress, inflammation, protein accumulation (amyloid and tau), and brain atrophy seem to be the main physiological mechanisms that lead to this association [117,253]. Regarding specific food intake, a recent meta-analysis found a significant linear relationship between fish consumption and the reduced risk of dementia [254]. In this sense, omega-3 (monounsaturated fatty acids) and polyunsaturated fatty acids (especially n3-PUFA) have been associated with a protective effect against dementia/AD [216,255,256]. Moreover, some antioxidants such as Vitamin E have also been linked to a protective effect against AD [257]. Finally, a recent meta-analysis has provided clear evidence that low levels of Vitamin D (25-hydroxyvitamin D) are associated with increased cognitive decline and AD [258,259,260,261,262]. This vitamin, present in some foods and synthesized in the skin by sun exposure, plays a significant role in vascular and immune systems.

#### 4.6.4. Physical Activity (PA)

It is well-known that regular exercise reduces the risk of dementia [15,112,263,264,265], whereas poor physical activity (PA) may be responsible for 13% of AD worldwide cases [130]. While the benefits of PA in brain health have been well-documented, the influence of sedentarism remains to be understood [266]. Basically, regular exercise provides numerous benefits to physical and mental health, including the prevention of metabolic and cardiovascular diseases, obesity, inflammatory processes, hormonal disequilibrium, and depression, which may exert a significant influence on individuals’ health. In addition, PA is also associated with larger brain volumes, specifically in brain regions vulnerable to dementia and improvements in brain connectivity [267,268]. PA increases the production of neurotrophic molecules (e.g., BDNF) and reduces the expression of other molecules associated with neuropathology (amyloid and/or tau). These changes support processes associated with better brain health (neurogenesis, angiogenesis) compared to other factors that have a negative influence (e.g., inflammation or oxidative stress) [268].

#### 4.6.5. Social Isolation

Recent studies indicate that social isolation increases the risk of dementia by 28% [269]. Individuals who live alone have less cognitive stimulation, making them more susceptible to early cognitive decline [269,270]. Moreover, unmarried men have a higher risk of dementia, although this relationship was not confirmed in women [271]. Likewise, widows are particularly susceptible to developing AD [272], with a higher risk of dementia in those without offspring [273]. According to some researchers, the feeling of loneliness rather than the social network size determines the increased dementia risk [274]. Social isolation has been associated with AD biomarkers such as amyloid and tau deposition [275]. Otherwise, it may increase the occurrence of collateral risk dementia/AD factors such as depression, anxiety, VRFs, reduced cognitive activity, or the failure to benefit from social resources (e.g., information and health-care access).

### 4.7. Population Factors

#### 4.7.1. Air Pollution

There is consistent evidence that air pollutants increase the risk of dementia and cause cognitive decline [15,276,277,278,279,280]. Basically, air pollution exposure, especially to fine particulate matter, may increase the risk of hypertension, lipid accumulation, atherosclerosis, oxidative stress, insulin resistance, endothelial dysfunction, propensity toward blood coagulation, inflammation, and stroke, all of which are related to an increased risk of dementia [281].

#### 4.7.2. Other Population Risk Factors

Exposure to pesticides or heavy metals may increase dementia risk by up to 50% [138,144]. Additional exposures, including other metals, solvents, or electromagnetic fields, might contribute to increased dementia risk, although scientific evidence remains inconclusive [138]. It is also worth noting that unfavorable socioeconomic statuses (e.g., unemployment, lower income) have been significantly associated with an increased risk of developing AD [17]. In brief, exposure to unfavorable environmental conditions can impact health status and increase the risk of dementia/AD, but future investigations should elucidate how these factors are related to different dementia subtypes.

## 5. Implications of Modifiable Risk Factors for Intervention Programs

Our knowledge about modifiable factors should ultimately lead to implementing prevention strategies and targeted intervention programs aimed at reducing dementia/AD risk. Basically, it is essential to consider that dementia is a complex, multifactorial heterogeneous syndrome, and preventive interventions should be focused on targeting several risk factors [282]. Accordingly, Ritchie et al. (2010) [129] published the first attempt to model the effects of a theoretical population-wide prevention strategy (ESPRIT study), comparing the relative effect of removing risk exposures over 7 years. In the absence of effective treatments, the authors underlined the importance of multicomponent health population-based plans aimed at reducing cognitive impairment and dementia RFs (i.e., control of diabetes, depression, high blood pressure, and CVD, fruit, vegetable and fish consumption, enhancement of crystallized intelligence, promotion of physical exercise),. According with this idea, the FINGER study [19,283], a pioneering long-term randomized controlled trial, examined a multimodal program of preventive interventions in 1260 subjects at risk of dementia from the general population. Six hundred thirty-one participants were randomly assigned to a program that included diet, exercise, cognitive training, and vascular risk management. After 2 years, the study showed that the intervention significantly benefited the global cognitive performance (main outcome) regardless of participants’ baseline characteristics. Other similar studies, such as the French MAPT trial [284] and the Dutch PreDIVA trial [285], designed with the same objectives, could not achieve similar cognitive results.

Recent reviews and meta-analyses have grouped RFs considering specific preventive strategies (i.e., targeting the body, compensatory interventions for brain aging, and health promotion) [284]. Particularly, Hussenoeder et al. (2018) [282] proposed a public brain health agenda following 10 key actions: (1) increasing physical activity, (2) fostering social integration, (3) improving education and fostering lifelong learning, (4) providing mentally stimulating workplaces, (5) fostering a cognitively active lifestyle, (6) proposing a Mediterranean-like diet, (7) reducing alcohol consumption, (8) stop smoking, (9) managing chronic conditions, and (10) reducing anticholinergic medication. A major challenge in establishing the effectiveness of these interventions is the limited number of long-term clinical trials, selection bias, and difficulty controlling for confounding variables. Despite this, it is essential to develop collaborative efforts to enhance new multicomponent intervention models to prevent cognitive decline and dementia at population and individual levels.

## 6. Discussion

Since dementia is a complex and multifactorial condition, genetic and lifestyle factors, environmental exposures, and diverse medical conditions may increase the associated risk due to synergistic and complex interactive effects. Basically, it is known that autosomal cases (i.e., presenilin-1 and 2, APP) linked to AD, the most common cause of dementia, represent a very small percentage of AD cases (≤1%) versus those with a sporadic late-onset disease [286]. In fact, new investigations are covering more than 300 genes related to AD [287]. Furthermore, environmental factors may play an important role in sporadic cases, explaining between 40 and 60% of the variability [15,114]. Within this framework, the epigenetic hypothesis claims that dementia is not a suddenly occurring state, but rather a gradual change in the cellular activity that finally impacts on the baseline healthy status [288]. Thus, accumulated environmental hits may produce latent epigenetic changes (e.g., DNA methylation), altering biochemical pathways/mechanisms until a pathological threshold is reached and clinical dementia becomes apparent. It is noteworthy that dementia/AD syndrome results from numerous brain pathologies [289], involving complex neurobiological mechanisms [290].

Different scientific reports have shown that the incidence of dementia/AD among older adults over the past two decades has declined in developed Western countries. However, further evidence is needed to prove this in non-Western and developing countries. Apparently, improvements in education, lifestyle, and public health may underlie these observations, but the explanation of this enigma is controversial. The Lancet Commission had proposed 12 main modifiable risk factors associated with dementia at different weights (range: 1–8%, with education and hearing loss being the highest versus alcohol, obesity, and diabetes as the lowest) and life periods [15]. However, other reports extend them to over 200, and the Manifesto of Berlin indicates that 35% of dementia cases could be avoided just by preventing stroke and VRFs [17,118]. In fact, the precise weight of each RFs on dementia risk is difficult to establish across different worldwide populations, and there are many questions about how the RFs and PFs exert their effect. For instance, it remains unclear which type of PA (intensity, frequency and duration) offer the greatest protective effect. Different studies have also pointed out the benefits of the Mediterranean diet on brain and cognitive functioning, but there is no universal diet pattern, and how these patterns affect the brain remains undetermined. Lastly, it is necessary to understand better the interplay between socio-behavioral habits and individual dementia RFs. Indeed, Knopman et al. (2010) [291] have pointed out that the genesis of VRFs has its roots in childhood social class, education, and culture.

**Figure 2:**
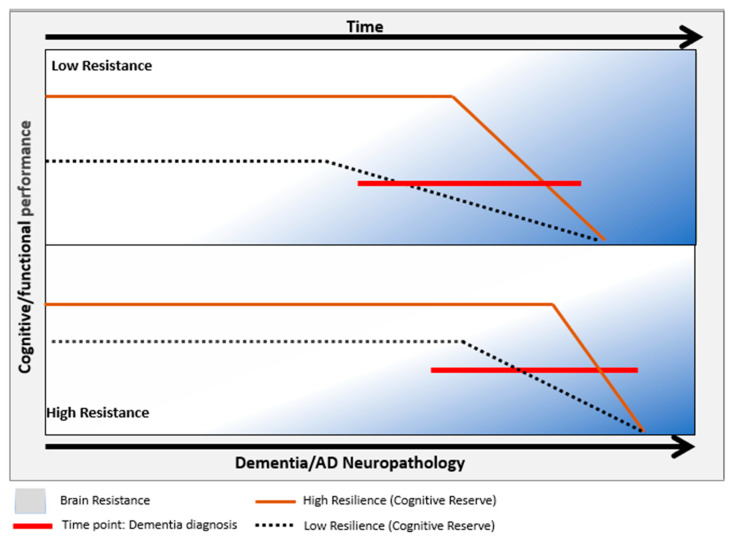
Coping against dementia: resilience and resistance mechanisms. The term resistance implies slower or delaying the onset of AD-associated neuropathology represented by blue color. However, resilience refers to the brain’s capacity to maintain cognitive and functional performance against pathology, with cognitive reserve being the most known form. Thus, people with higher CR (brown line) may show better performance than those with lower CR when facing similar levels of pathology.

At this point, it is worth considering whether dementia can be avoided. A recent robust meta-analyses (4677 subjects from 17 population-based cohorts) report that one-third of community-dwelling older adults with intermediate–high levels of AD neuropathology are not clinically demented [292]. Furthermore, some individuals reach the age of 100 having preserved their cognitive abilities, showing that exceptional longevity may be associated with neuroprotection mechanisms: resistance (i.e., avoiding pathologies) and resilience (i.e., coping with pathologies) [293,294]. These general terms (see Figure 2) are complementary and comprise specific underlying processes that are operationalized in detail elsewhere [295,296,297]. Basically, resilience entails three mechanisms: Brain Reserve (BR), Cognitive Reserve (CR), and Brain Maintenance (BM). BR is conceived as neurobiological capital (i.e., neurons, synapses). Therefore, some authors indicate that individuals with a more significant number of neurons and synapses may withstand a more significant neuropathological load before manifesting dementia symptoms [298]; CR refers to the active capacity of the brain to deal with brain damage, essentially activating compensation mechanisms (i.e., alternative brain networks and/or cognitive strategies) [299]. Finally, BM is defined as reduced development of age-related brain changes and pathology to preserve cognitive abilities. Higher resistance to the progression of neuropathology may be a form of BM. In brief, resilience describes individual differences in terms of brain structure and functioning, including compensation processes, based on genetics or lifestyle factors (e.g., cognitive stimulating activities). In general, the brain is plastic and can accumulate capacities and/or resources over time to cope with adverse situations. Otherwise, resistance mainly refers to the absence or lower rates than expected of pathology. This terminology is useful for communication across investigators, but more consensus is required in terms of terminology and operationalization [300].

The primary limitations of this review should be considered. First, this review was not systematic, and the selection of information was based on the authors’ expertise, facilitating a potential selection bias. However, it is remarkable that we covered a comprehensive number of high-quality publications (i.e., longitudinal population-based cohorts, systematic reviews, and meta-analysis) related to dementia/AD worldwide. Second, most studies evaluate the specific risk of dementia, rather than AD, in specific countries/populations. Moreover, there are significant differences in terms of methodology between studies (e.g., intervals, statistical analyses), which were not specifically addressed in this review. These aspects invite caution about the generalization of results.

## 7. Conclusions

Currently, scientific evidence suggests that dementia/AD is preventable, but there is a lack of consensus on the potential extent of prevention and no sure way to prevent all types of dementia exists. Accordingly, well-designed longitudinal population-based cohorts, across different populations, are needed to chart the specific weights of dementia/AD risk factors and their complex interactions, underpinning their specific mechanisms of action. Thus, it is mandatory to investigate further how environmental exposures impact our organic systems, in particular the genes and brain, across different life periods. The future of prevention comprises the development of tailored interventions and, especially, public health strategies. The design of specific preventive strategies by dementia subtypes is a present challenge in the field. Finally, dementia requires a broad and coordinated social response, designing policies to promote preventive strategies at a population-based level and high-risk groups. The development of comprehensive programs, through a coordinated and multisectoral approach in each country, is a promising perspective for preventing dementia in the future.

## Figures and Tables

**Table 1:** Incidence studies of dementia/AD studies with long-term follow-up intervals.

Author/Year/Country	Follow-Up (Years)	Dementia Subtype	Preval	Incidence	Study Name
Western countries					
Manton/2005/US [25] **	18	Mixed	↓	—	LNLTCS
Langa/2008/US [33] *	9	Dementia	↓	—	HRS
Hall/2009/US [34] *	9	Dementia	#	—	Indianapolis cohort. (African Americans)
Hebert/2010/US [35] *	10	AD	—	#	Chicago neighborhoods
Lobo/2011/Spain [36] *	>10	Dementia	↓	— α	ZARADEMP
Rocca/2011/US [26] **	10–20	Dem/AD	—	↓ #	Several
Schrijvers/2012/Holland [37] *	10	Dem	—	↓ &	Rotterdam
Wiberg/2013/Sweden [38] *	30	Dementia	#	—	Gothenburg cohorts
Abdulrahman/2014/UK [39] **	12	AD	↑	↑	PEDW (Wales)
Grasset/2016/France [40] *	10	Dementia	—	↓ ω	PAQUID-Three City
Matthews/2016/UK [41] *	7–12	Dementia	—	↓	CFAS
Satizabal/2016//US [3] *	30	Dementia	—	↓	Framingham Heart
Kosteniuk/2016/Canada [42] **	8	Dementia	↑	↓	Saskatchewan heath data
Wimo/2016/Sweden [43] *	6	Dementia	↓	—	Sweden, rural area
Ahmadi/2017/UK [44] **	10	Dementia	—	↓	ELSA
Cerasuolo/2017/Canada [45]	12	Dementia	—	↓ ~	Several
Derby/2017/US [34] *	22	Dementia	—	↓	Einstein Aging Study
Noble/2017/US [46] *	7	Dementia	—	↓	WH-I-Aging Study
Peres/2017/France [47] *	20	Dementia	↓	—	PAQUID&AMI
Chen/2018/US [48] **	12	Dementia	↓	—	HR Study
Hendrie/2018/US [49] *	9	Dem/AD	—	↓	WHICAP
Seblova/2018/Sweden [50] **	30	Dementia	—	↓	National Swedish Registry
Rajan/2018/US [51]	18	AD	#	#	CHAP Study
Sullivan/2019/US [52] *	40	Dementia	—	↓	Monongahela Valley
Ding/2020/Sweden [53] *	25	Dementia	—	↓	Stockholm (2 Cohorts)
Wolters/2020/US-Europe [12] *	27	Dem/AD	—	↓	Several
Farina/2022/US [54] **	16	Dem/AD	↓	↓	HRS
Van Bussell/2022/Netherland [55]	12	Dementia	—	#	Dutch Primary Care
Chen-Y-/2023/UK [56]	17	Dementia	—	↓γ, ↑γ	ELS of Aging
Non-Western countries					
Gao/2016/Nigeria [57]	9	Dem/AD	—	#	IIDP
Ohara/2017/Japan [58]	27	Dem/AD	↑	↑	Hisayama
Ding/2020/China [59]	29	Dementia	↑	↑	SESD&SAS (Shanghai)
Shimizu/2022/Japan [60] *	19	Dem/AD	↑	—	Nayakama town
Huang/2024/Taiwan [61]	13	Dementia	—	↑	National Taiwan cohort

All included studies have at least 5 years of follow-up and data are referred to the last waves. ↓ Decrease; ↑ Increase; # Stable; — Not studied; & without statistical significance; * Population cohorts; ** Database, cohorts; α: Only in men; ω: Only in women; ~: only in very old; #: only shown in a group. ↓γ period 2002–2010, ↑γ period 2010–2019. Abbreviations: Preval: Prevalence; Dem = Dementia; UK: United Kingdom; US: United States. Study names: CFAS: Cognitive Function and Ageing; CHAP: Chicago Health and Aging Population; ELSA: English Longitudinal Study of Ageing; HRS: Health and Retirement Study; IIDP: Indianapolis-Ibadan project; LNLTCS: National Long Term Care Surveys; PAQUID: Personnes Agés QUID; PEDW: Patient Episode Database for Wales; SEDS: Shanghai Epidemiological Survey of Dementia and Alzheimer’s disease; WHICAP: Washington Heights-Inwood Columbia Aging; ZARADEMP: Dementia Prevalence Study from Saragossa, Spain.

**Table 2:** Selection of reviews on dementia/Alzheimer’s disease risk factors.

Author/Publication Year	Risk Factors	Dementia Outcome	Preventable %, Study Type
Henderson, (1988) [115]	CRF, SERF, environmental RF	AD	-
EURODEM group [116]	Clinical and environmental RF	AD	
EURODEM group [125]	Lifestyle RF	AD	
Haan and Wallace (2004) [126]	VRF, genetics, and exposure RF	AD/VaD	
Jansson (2005) [127]	Clinical and biological RF	AD	50%
Middleton and Yaffe (2009) [128]	CRF and lifestyle	Dementia	
Ritchie et al. (2010) [129]	CRF	Dementia	Specific % by RFs
Barnes and Jaffe (2011) [130]	7 CRF	EA	up to 50.7% ¥
Song et al. (2011) [131]	Frailty index	Dementia/AD	-
Mangialasche et al. (2012) [23]	Clinical and lifestyle RF	Dementia/AD/VaD	
Anstey et al. (2013) [132]	11 RF and 4 PF	AD	
Di Marco et al. (2014) [133]	Modifiable lifestyle RF	Dementia	Systematic Review of Cohorts
Anstey et al. (2015) [134]	CRF and lifestyle RF	Dementia	-
Baumgart et al. (2015) [135]	CRF and lifestyle RF	Dementia	
Deckers et al. (2015) [136]	CRF (midlife)	Dementia/MCI	Expert Delphi Panel
Xu et al. (2015) [114]	93 clinical, lifestyle, exposure RF	AD	66%
Hazar et al. (2016) [137]	5 CRF	AD	33%
Killin et al. (2016) [138]	Environmental RF	Dementia	
Wu et al. (2016) [139]	11 RF	Dementia	
Bellou et al. (2017) [140]	Environmental RF and CRF	Dem/AD/VaD	
Livingston et al. (2017) [112]	9 CRF	Dementia	
Rakesh et al. (2017) [141]	Multiples CRF and lifestyle RF	Dementia	
Larsson and Markus, (2018) [142]	VRF	Dementia/AD	Systematic Review & MA
Anstey et al. (2019) [143]	RF: AD 34; Dem 69, VaD 8	AD/Dem/VaD	
Armstrong (2019) [144]	51 RF	AD	
Edwards III GA et al. (2019) [145]	CRF and lifestyle RF	AD	
Peters et al. (2019) [146]	Co-ocurring RF	Dementia	Systematic Review & MA
Rochoy et al. (2019) [147]	Multiples RF	AD	
Yu et al. (2020) [148]	104 modifiable RF, 11 interventions	AD	
Liang et al. (2020) [149]	Modifiable RF	Dementia	Bayesian Analysis & MA
Livingston et al. (2020) [15]	12 CRF	Dementia	40%
Kuo et al. (2020) [150]	Modifiable/Non-modifiable RF	Dementia/AD	Review
Rolandi et al. (2020) [151]	Modifiable FR cohort	Dementia	40%
Weiss et al. (2020) [16]	65 RF	Dementia	
Zhang et al. (2023) [17]	210 CRF, lifestyle, SERF	Dementia	47.0–72.6%
Jones et al. (2024) [152]	14 Modifiable RF	AD/VaD	Umbrella-Review & MA
Stephan et al. (2024) [153]	Modifiable RF	Dementia	Systematic Review & MA

Abbreviations: RF: Risk Factor; PF: Protective Factor; CRF: Clinical Risk Factor; VRF: Vascular Risk Factors. VaD: Vascular Dementia; AD: Alzheimer’s Disease. SERF; socioeconomic risk factors; MA: meta-analysis. Preventable %: Percentage of Preventable Dementia; ¥ Various risk factors are reduced by 10–25%.

## Abbreviations

Aβ	Amyloid Beta
AD	Alzheimer’s Disease
ApoE	Apolipoprotein E
CHAP	Chicago Health and Aging Project
CFAS	Cognitive Function and Ageing Study
CVD	Cardiovascular Disease
DM	Diabetes Mellitus
DOHaD	Developmental Origins of Health and Disease
GWAS	Genome-Wide Association Studies
IIDP	Indianapolis-Ibadan Dementia Project
MCI	Mild Cognitive Impairment
NICE	National Institute for Health and Care Excellence
PA	Physical Activity
PRS	Polygenic Risk Score
RF	Risk Factors
SAS	Survey of Aging Shanghai
SESD	Shanghai Epidemiological Survey of Dementia
SNPs	Single Nucleotide Polymorphisms
TBI	Traumatic Brain Injury
VaD	Vascular Dementia
VRFs	Vascular Risk Factors
WHO	World Health Organization

## References

[1] Wimo A., Seeher K., Cataldi R., Cyhlarova E., Dielemann J.L., Frisell O., Guerchet M., Jönsson L., Malaha A.K., Nichols E. (2023). The Worldwide Costs of Dementia in 2019. Alzheimers Dement..

[2] Gustavsson A., Norton N., Fast T., Frölich L., Georges J., Holzapfel D., Kirabali T., Krolak-Salmon P., Rossini P.M., Ferretti M.T. (2023). Global Estimates on the Number of Persons across the Alzheimer’s Disease Continuum. Alzheimers Dement..

[3] Satizabal C.L., Beiser A.S., Chouraki V., Chêne G., Dufouil C., Seshadri S. (2016). Incidence of Dementia over Three Decades in the Framingham Heart Study. N. Engl. J. Med..

[4] World Health Organization (2017). Global Action Plan on the Public Health Response to Dementia 2017–2025.

[5] World Health Organization (2019). Risk Reduction of Cognitive Decline and Dementia: WHO Guidelines.

[6] Arnsten A.F.T., Datta D., Del Tredici K., Braak H. (2021). Hypothesis: Tau Pathology Is an Initiating Factor in Sporadic Alzheimer’s Disease. Alzheimers Dement..

[7] Braak H., Thal D.R., Ghebremedhin E., Del Tredici K. (2011). Stages of the Pathologic Process in Alzheimer Disease: Age Categories from 1 to 100 Years. J. Neuropathol. Exp. Neurol..

[8] Braak H., Del Tredici K. (2012). Where, When, and in What Form Does Sporadic Alzheimer’s Disease Begin?. Curr. Opin. Neurol..

[9] Khachaturian Z.S., Khachaturian A.S. (2015). Politics of Science: Progress toward Prevention of the Dementia–Alzheimer’s Syndrome. Mol. Asp. Med..

[10] Nichols E., Steinmetz J.D., Vollset S.E., Fukutaki K., Chalek J., Abd-Allah F., Abdoli A., Abualhasan A., Abu-Gharbieh E., Akram T.T. (2022). Estimation of the Global Prevalence of Dementia in 2019 and Forecasted Prevalence in 2050: An Analysis for the Global Burden of Disease Study 2019. Lancet Public Health.

[11] Roehr S., Pabst A., Luck T., Riedel-Heller S. (2018). Is Dementia Incidence Declining in High-Income Countries? A Systematic Review and Meta-Analysis. Clin. Epidemiol..

[12] Wolters F.J., Chibnik L.B., Waziry R., Anderson R., Berr C., Beiser A., Bis J.C., Blacker D., Bos D., Brayne C. (2020). Twenty-Seven-Year Time Trends in Dementia Incidence in Europe and the United States: The Alzheimer Cohorts Consortium. Neurology.

[13] Wu Y.-T., Beiser A.S., Breteler M.M.B., Fratiglioni L., Helmer C., Hendrie H.C., Honda H., Ikram M.A., Langa K.M., Lobo A. (2017). The Changing Prevalence and Incidence of Dementia over Time—Current Evidence. Nat. Rev. Neurol..

[14] Knopman D.S. (2020). The Enigma of Decreasing Dementia Incidence. JAMA Netw. Open.

[15] Livingston G., Huntley J., Sommerlad A., Ames D., Ballard C., Banerjee S., Brayne C., Burns A., Cohen-Mansfield J., Cooper C. (2020). Dementia Prevention, Intervention, and Care: 2020 Report of the Lancet Commission. Lancet.

[16] Weiss J., Puterman E., Prather A.A., Ware E.B., Rehkopf D.H. (2020). A Data-Driven Prospective Study of Dementia among Older Adults in the United States. PLoS ONE.

[17] Zhang Y., Chen S.-D., Deng Y.-T., You J., He X.-Y., Wu X.-R., Wu B.-S., Yang L., Zhang Y.-R., Kuo K. (2023). Identifying Modifiable Factors and Their Joint Effect on Dementia Risk in the UK Biobank. Nat. Hum. Behav..

[18] Ngandu T., Lehtisalo J., Solomon A., Levälahti E., Ahtiluoto S., Antikainen R., Bäckman L., Hänninen T., Jula A., Laatikainen T. (2015). A 2 Year Multidomain Intervention of Diet, Exercise, Cognitive Training, and Vascular Risk Monitoring versus Control to Prevent Cognitive Decline in at-Risk Elderly People (FINGER): A Randomised Controlled Trial. Lancet.

[19] Rosenberg A., Mangialasche F., Ngandu T., Solomon A., Kivipelto M. (2020). Multidomain Interventions to Prevent Cognitive Impairment, Alzheimer’s Disease and Dementia: From Finger to World-Wide Fingers. J. Prev. Alzheimers Dis..

[20] Dacks P.A., Andrieu S., Blacker D., Carman A.J., Green A.M., Grodstein F., Henderson V.W., James B.D., Lane R.F., Lau J. (2014). Dementia Prevention: Optimizing the Use of Observational Data for Personal, Clinical, and Public Health Decision-Making. J. Prev. Alzheimers Dis..

[21] Eggink E., Moll Van Charante E.P., Van Gool W.A., Richard E. (2019). A Population Perspective on Prevention of Dementia. J. Clin. Med..

[22] Frisoni G.B., Altomare D., Ribaldi F., Villain N., Brayne C., Mukadam N., Abramowicz M., Barkhof F., Berthier M., Bieler-Aeschlimann M. (2023). Dementia Prevention in Memory Clinics: Recommendations from the European Task Force for Brain Health Services. Lancet Reg. Health Eur..

[23] Mangialasche F., Kivipelto M., Solomon A., Fratiglioni L. (2012). Dementia Prevention: Current Epidemiological Evidence and Future Perspective. Alzheimers Res. Ther..

[24] Maresova P., Rezny L., Bauer P., Valko M., Kuca K. (2024). Nonpharmacological Intervention Therapies for Dementia: Potential Break-Even Intervention Price and Savings for Selected Risk Factors in the European Healthcare System. BMC Public Health.

[25] Manton K.C., Gu X.L., Ukraintseva S.V. (2005). Declining Prevalence of Dementia in the U.S. Elderly Population. Adv. Gerontol. Uspekhi Gerontol..

[26] Rocca W.A., Petersen R.C., Knopman D.S., Hebert L.E., Evans D.A., Hall K.S., Gao S., Unverzagt F.W., Langa K.M., Larson E.B. (2011). Trends in the Incidence and Prevalence of Alzheimer’s Disease, Dementia, and Cognitive Impairment in the United States. Alzheimers Dement..

[27] Mayer F., Remoli G., Bacigalupo I., Palazzesi I., Piscopo P., Bellomo G., Canevelli M., Corbo M., Vanacore N., Lacorte E. (2021). Decreasing Trend in the Incidence and Prevalence of Dementia: A Systematic Review. Minerva Med..

[28] Morovatdar N., Avan A., Azarpazhooh M.R., Di Napoli M., Stranges S., Kapral M.K., Rezayat A.A., Shariatzadeh A., Abootalebi S., Mokhber N. (2022). Secular Trends of Ischaemic Heart Disease, Stroke, and Dementia in High-Income Countries from 1990 to 2017: The Global Burden of Disease Study 2017. Neurol. Sci..

[29] Okamura H., Ishii S., Ishii T., Eboshida A. (2013). Prevalence of Dementia in Japan: A Systematic Review. Dement. Geriatr. Cogn. Disord..

[30] Kim Y.J., Han J.W., So Y.S., Seo J.Y., Kim K.Y., Kim K.W. (2014). Prevalence and Trends of Dementia in Korea: A Systematic Review and Meta-Analysis. J. Korean Med. Sci..

[31] Zhu Y., Liu H., Lu X.-L., Zhang B., Weng W., Yang J., Zhang J., Dong M.-J. (2019). Prevalence of Dementia in the People’s Republic of China from 1985 to 2015: A Systematic Review and Meta-Regression Analysis. BMC Public Health.

[32] Ribeiro F., Teixeira-Santos A.C., Caramelli P., Leist A.K. (2022). Prevalence of Dementia in Latin America and Caribbean Countries: Systematic Review and Meta-Analyses Exploring Age, Sex, Rurality, and Education as Possible Determinants. Ageing Res. Rev..

[33] Langa K.M., Larson E.B., Karlawish J.H., Cutler D.M., Kabeto M.U., Kim S.Y., Rosen A.B. (2008). Trends in the Prevalence and Mortality of Cognitive Impairment in the United States: Is There Evidence of a Compression of Cognitive Morbidity?. Alzheimers Dement..

[34] Derby C.A., Katz M.J., Lipton R.B., Hall C.B. (2017). Trends in Dementia Incidence in a Birth Cohort Analysis of the Einstein Aging Study. JAMA Neurol..

[35] Hebert L.E., Bienias J.L., Aggarwal N.T., Wilson R.S., Bennett D.A., Shah R.C., Evans D.A. (2010). Change in Risk of Alzheimer Disease over Time. Neurology.

[36] Lobo A., Lopez-Anton R., Santabárbara J., de-la-Cámara C., Ventura T., Quintanilla M.A., Roy J.F., Campayo A.J., Lobo E., Palomo T. (2011). Incidence and Lifetime Risk of Dementia and Alzheimer’s Disease in a Southern European Population: Incidence and LTR of Dementia and AD. Acta Psychiatr. Scand..

[37] Schrijvers E.M.C., Verhaaren B.F.J., Koudstaal P.J., Hofman A., Ikram M.A., Breteler M.M.B. (2012). Is Dementia Incidence Declining? Trends in Dementia Incidence since 1990 in the Rotterdam Study. Neurology.

[38] Wiberg P., Waern M., Billstedt E., Östling S., Skoog I. (2013). Secular Trends in the Prevalence of Dementia and Depression in Swedish Septuagenarians 1976–2006. Psychol. Med..

[39] Abdulrahman G.O. (2014). Alzheimer’s Disease: Current Trends in Wales. Oman Med. J..

[40] Grasset L., Brayne C., Joly P., Jacqmin-Gadda H., Peres K., Foubert-Samier A., Dartigues J., Helmer C. (2016). Trends in Dementia Incidence: Evolution over a 10-year Period in France. Alzheimers Dement..

[41] Matthews F.E., Stephan B.C.M., Robinson L., Jagger C., Barnes L.E., Arthur A., Brayne C., Comas-Herrera A., Wittenberg R., Cognitive Function and Ageing Studies (CFAS) Collaboration (2016). A Two Decade Dementia Incidence Comparison from the Cognitive Function and Ageing Studies I and II. Nat. Commun..

[42] Kosteniuk J.G., Morgan D.G., O’Connell M.E., Kirk A., Crossley M., Teare G.F., Stewart N.J., Bello-Haas V.D., McBain L., Mou H. (2016). Simultaneous Temporal Trends in Dementia Incidence and Prevalence, 2005–2013: A Population-Based Retrospective Cohort Study in Saskatchewan, Canada. Int. Psychogeriatr..

[43] Wimo A., Sjölund B.-M., Sköldunger A., Qiu C., Klarin I., Nordberg G., Von Strauss E. (2016). Cohort Effects in the Prevalence and Survival of People with Dementia in a Rural Area in Northern Sweden. J. Alzheimers Dis..

[44] Ahmadi-Abhari S., Guzman-Castillo M., Bandosz P., Shipley M.J., Muniz-Terrera G., Singh-Manoux A., Kivimäki M., Steptoe A., Capewell S., O’Flaherty M. (2017). Temporal Trend in Dementia Incidence since 2002 and Projections for Prevalence in England and Wales to 2040: Modelling Study. BMJ.

[45] Cerasuolo J.O., Cipriano L.E., Sposato L.A., Kapral M.K., Fang J., Gill S.S., Hackam D.G., Hachinski V. (2017). Population-based Stroke and Dementia Incidence Trends: Age and Sex Variations. Alzheimers Dement..

[46] Noble J.M., Schupf N., Manly J.J., Andrews H., Tang M.-X., Mayeux R. (2017). Secular Trends in the Incidence of Dementia in a Multi-Ethnic Community. J. Alzheimers Dis..

[47] Pérès K., Brayne C., Matharan F., Grasset L., Helmer C., Letenneur L., Foubert-Samier A., Baldi I., Tison F., Amieva H. (2017). Trends in Prevalence of Dementia in French Farmers from Two Epidemiological Cohorts. J. Am. Geriatr. Soc..

[48] Chen C., Zissimopoulos J.M. (2018). Racial and Ethnic Differences in Trends in Dementia Prevalence and Risk Factors in the United States. Alzheimers Dement. Transl. Res. Clin. Interv..

[49] Hendrie H.C., Smith-Gamble V., Lane K.A., Purnell C., Clark D.O., Gao S. (2018). The Association of Early Life Factors and Declining Incidence Rates of Dementia in an Elderly Population of African Americans. J. Gerontol. Ser. B.

[50] Seblova D., Quiroga M., Fors S., Johnell K., Lövdén M., Ponce De Leon A., Svensson A., Wicks S., Lager A. (2018). Thirty-Year Trends in Dementia: A Nationwide Population Study of Swedish Inpatient Records. Clin. Epidemiol..

[51] Rajan K.B., Weuve J., Barnes L.L., Wilson R.S., Evans D.A. (2019). Prevalence and Incidence of Clinically Diagnosed Alzheimer’s Disease Dementia from 1994 to 2012 in a Population Study. Alzheimers Dement..

[52] Sullivan K.J., Dodge H.H., Hughes T.F., Chang C.-C.H., Zhu X., Liu A., Ganguli M. (2019). Declining Incident Dementia Rates Across Four Population-Based Birth Cohorts. J. Gerontol. Ser. A.

[53] Ding M., Qiu C., Rizzuto D., Grande G., Fratiglioni L. (2020). Tracing Temporal Trends in Dementia Incidence over 25 Years in Central Stockholm, Sweden. Alzheimers Dement..

[54] Farina M.P., Zhang Y.S., Kim J.K., Hayward M.D., Crimmins E.M. (2022). Trends in Dementia Prevalence, Incidence, and Mortality in the United States (2000–2016). J. Aging Health.

[55] Van Bussel E.F., Richard E., Arts D.L., Nooyens A.C.J., Coloma P.M., De Waal M.W.M., Van Den Akker M., Biermans M.C.J., Nielen M.M.J., Van Boven K. (2017). Dementia Incidence Trend over 1992–2014 in the Netherlands: Analysis of Primary Care Data. PLOS Med..

[56] Chen Y., Bandosz P., Stoye G., Liu Y., Wu Y., Lobanov-Rostovsky S., French E., Kivimaki M., Livingston G., Liao J. (2023). Dementia Incidence Trend in England and Wales, 2002–2019, and Projection for Dementia Burden to 2040: Analysis of Data from the English Longitudinal Study of Ageing. Lancet Public Health.

[57] Gao S., Ogunniyi A., Hall K.S., Baiyewu O., Unverzagt F.W., Lane K.A., Murrell J.R., Gureje O., Hake A.M., Hendrie H.C. (2016). Dementia Incidence Declined in African-Americans but Not in Yoruba. Alzheimers Dement..

[58] Ohara T., Hata J., Yoshida D., Mukai N., Nagata M., Iwaki T., Kitazono T., Kanba S., Kiyohara Y., Ninomiya T. (2017). Trends in Dementia Prevalence, Incidence, and Survival Rate in a Japanese Community. Neurology.

[59] Ding D., Zhao Q., Wu W., Xiao Z., Liang X., Luo J., Hong Z. (2020). Prevalence and Incidence of Dementia in an Older Chinese Population over Two Decades: The Role of Education. Alzheimers Dement..

[60] Shimizu H., Mori T., Yoshida T., Tachibana A., Ozaki T., Yoshino Y., Ochi S., Sonobe N., Matsumoto T., Komori K. (2022). Secular Trends in the Prevalence of Dementia Based on a Community-based Complete Enumeration in Japan: The Nakayama Study. Psychogeriatrics.

[61] Huang S.-T., Loh C.-H., Lin C.-H., Hsiao F.-Y., Chen L.-K. (2024). Trends in Dementia Incidence and Mortality, and Dynamic Changes in Comorbidity and Healthcare Utilization from 2004 to 2017: A Taiwan National Cohort Study. Arch. Gerontol. Geriatr..

[62] Mahmood S.S., Levy D., Vasan R.S., Wang T.J. (2014). The Framingham Heart Study and the Epidemiology of Cardiovascular Disease: A Historical Perspective. Lancet.

[63] Brandes N., Weissbrod O., Linial M. (2022). Open Problems in Human Trait Genetics. Genome Biol..

[64] Bergem A.L.M. (1997). The Role of Heredity in Late-Onset Alzheimer Disease and Vascular Dementia: A Twin Study. Arch. Gen. Psychiatry.

[65] Nordestgaard L.T., Christoffersen M., Frikke-Schmidt R. (2022). Shared Risk Factors between Dementia and Atherosclerotic Cardiovascular Disease. Int. J. Mol. Sci..

[66] Turnpenny P.D., Ellard S., Cleaver R. (2022). Emery’s Elements of Medical Genetics and Genomics.

[67] Wingo T.S. (2012). Autosomal Recessive Causes Likely in Early-Onset Alzheimer Disease. Arch. Neurol..

[68] Seto M., Weiner R.L., Dumitrescu L., Hohman T.J. (2021). Protective Genes and Pathways in Alzheimer’s Disease: Moving towards Precision Interventions. Mol. Neurodegener..

[69] Fortea J., Pegueroles J., Alcolea D., Belbin O., Dols-Icardo O., Vaqué-Alcázar L., Videla L., Gispert J.D., Suárez-Calvet M., Johnson S.C. (2024). APOE4 Homozygozity Represents a Distinct Genetic Form of Alzheimer’s Disease. Nat. Med..

[70] Arboleda-Velasquez J.F., Lopera F., O’Hare M., Delgado-Tirado S., Marino C., Chmielewska N., Saez-Torres K.L., Amarnani D., Schultz A.P., Sperling R.A. (2019). Resistance to Autosomal Dominant Alzheimer’s Disease in an APOE3 Christchurch Homozygote: A Case Report. Nat. Med..

[71] Baker E., Leonenko G., Schmidt K.M., Hill M., Myers A.J., Shoai M., De Rojas I., Tesi N., Holstege H., Van Der Flier W.M. (2023). What Does Heritability of Alzheimer’s Disease Represent?. PLoS ONE.

[72] Gatz M., Reynolds C.A., Fratiglioni L., Johansson B., Mortimer J.A., Berg S., Fiske A., Pedersen N.L. (2006). Role of Genes and Environments for Explaining Alzheimer Disease. Arch. Gen. Psychiatry.

[73] Migliore L., Coppedè F. (2022). Gene–Environment Interactions in Alzheimer Disease: The Emerging Role of Epigenetics. Nat. Rev. Neurol..

[74] Verheijen J., Sleegers K. (2018). Understanding Alzheimer Disease at the Interface between Genetics and Transcriptomics. Trends Genet..

[75] Dunn A.R., O’Connell K.M.S., Kaczorowski C.C. (2019). Gene-by-Environment Interactions in Alzheimer’s Disease and Parkinson’s Disease. Neurosci. Biobehav. Rev..

[76] Karlsson I.K., Escott-Price V., Gatz M., Hardy J., Pedersen N.L., Shoai M., Reynolds C.A. (2022). Measuring Heritable Contributions to Alzheimer’s Disease: Polygenic Risk Score Analysis with Twins. Brain Commun..

[77] Andrews S.J., Fulton-Howard B., Goate A. (2020). Interpretation of Risk Loci from Genome-Wide Association Studies of Alzheimer’s Disease. Lancet Neurol..

[78] Kunkle B.W., Schmidt M., Klein H.-U., Naj A.C., Hamilton-Nelson K.L., Larson E.B., Evans D.A., De Jager P.L., Crane P.K., Buxbaum J.D. (2021). Novel Alzheimer Disease Risk Loci and Pathways in African American Individuals Using the African Genome Resources Panel: A Meta-Analysis. JAMA Neurol..

[79] Pimenova A.A., Raj T., Goate A.M. (2018). Untangling Genetic Risk for Alzheimer’s Disease. Biol. Psychiatry.

[80] Lee S.H., Wray N.R., Goddard M.E., Visscher P.M. (2011). Estimating Missing Heritability for Disease from Genome-Wide Association Studies. Am. J. Hum. Genet..

[81] Bertram L., Tanzi R.E. (2020). Genomic Mechanisms in Alzheimer’s Disease. Brain Pathol..

[82] Bellenguez C., Küçükali F., Jansen I.E., Kleineidam L., Moreno-Grau S., Amin N., Naj A.C., Campos-Martin R., Grenier-Boley B., Andrade V. (2022). New Insights into the Genetic Etiology of Alzheimer’s Disease and Related Dementias. Nat. Genet..

[83] Gan J., Fu H., Zhu X. (2022). Relationships between Multiple Dimensions of Insight and Neurocognition, Metacognition, and Social Cognition: A Meta-Analysis. J. Nerv. Ment. Dis..

[84] Dattani S., Howard D.M., Lewis C.M., Sham P.C. (2022). Clarifying the Causes of Consistent and Inconsistent Findings in Genetics. Genet. Epidemiol..

[85] Choi S.W., Mak T.S.-H., O’Reilly P.F. (2020). Tutorial: A Guide to Performing Polygenic Risk Score Analyses. Nat. Protoc..

[86] Lourida I., Hannon E., Littlejohns T.J., Langa K.M., Hyppönen E., Kuzma E., Llewellyn D.J. (2019). Association of Lifestyle and Genetic Risk with Incidence of Dementia. JAMA.

[87] Ward D.D., Ranson J.M., Wallace L.M.K., Llewellyn D.J., Rockwood K. (2022). Frailty, Lifestyle, Genetics and Dementia Risk. J. Neurol. Neurosurg. Psychiatry.

[88] Marden J.R., Walter S., Tchetgen Tchetgen E.J., Kawachi I., Glymour M.M. (2014). Validation of a Polygenic Risk Score for Dementia in Black and White Individuals. Brain Behav..

[89] Jones L., Lambert J., Wang L., Choi S., Harold D., Vedernikov A., Escott-Price V., Stone T., Richards A., International Genomics of Alzheimer’s Disease Consortium (IGAP) (2015). Convergent Genetic and Expression Data Implicate Immunity in Alzheimer’s Disease. Alzheimers Dement..

[90] Naj A.C., Schellenberg G.D., for the Alzheimer’s Disease Genetics Consortium (ADGC) (2017). Genomic Variants, Genes, and Pathways of Alzheimer’s Disease: An Overview. Am. J. Med. Genet. B Neuropsychiatr. Genet..

[91] Ridge P.G., Hoyt K.B., Boehme K., Mukherjee S., Crane P.K., Haines J.L., Mayeux R., Farrer L.A., Pericak-Vance M.A., Schellenberg G.D. (2016). Assessment of the Genetic Variance of Late-Onset Alzheimer’s Disease. Neurobiol. Aging.

[92] Barker D.J.P., Osmond C., Winter P.D., Margetts B., Simmonds S.J. (1989). Weight in infancy and death from ischaemic heart disease. Lancet.

[93] Dover G.J. (2009). The Barker Hypothesis: How Pediatricans Will Diagnose and Prevent Common Adult-Onset Diseases. Trans. Am. Clin. Climatol. Assoc..

[94] Heindel J.J., Vandenberg L.N. (2015). Developmental Origins of Health and Disease: A Paradigm for Understanding Disease Cause and Prevention. Curr. Opin. Pediatr..

[95] Borenstein A.R., Copenhaver C.I., Mortimer J.A. (2006). Early-Life Risk Factors for Alzheimer Disease. Alzheimer Dis. Assoc. Disord..

[96] Bermejo-Pareja F. (2018). Alzheimer: Prevention from Childhood.

[97] Bleker L.S., De Rooij S.R., Painter R.C., Ravelli A.C., Roseboom T.J. (2021). Cohort Profile: The Dutch Famine Birth Cohort (DFBC)—A Prospective Birth Cohort Study in the Netherlands. BMJ Open.

[98] Melrose R.J., Brewster P., Marquine M.J., MacKay-Brandt A., Reed B., Farias S.T., Mungas D. (2015). Early Life Development in a Multiethnic Sample and the Relation to Late Life Cognition. J. Gerontol. B Psychol. Sci. Soc. Sci..

[99] Moody L., Chen H., Pan Y.-X. (2017). Early-Life Nutritional Programming of Cognition—The Fundamental Role of Epigenetic Mechanisms in Mediating the Relation between Early-Life Environment and Learning and Memory Process. Adv. Nutr..

[100] Fischer M., Lövdén M., Nilsson T., Seblova D. (2023). Very Early-Life Risk Factors for Developing Dementia: Evidence from Full Population Registers. J. Gerontol. Ser. B.

[101] Huang T.L., Carlson M.C., Fitzpatrick A.L., Kuller L.H., Fried L.P., Zandi P.P. (2008). Knee Height and Arm Span: A Reflection of Early Life Environment and Risk of Dementia. Neurology.

[102] Ruisch I.H., Dietrich A., Glennon J.C., Buitelaar J.K., Hoekstra P.J. (2018). Maternal Substance Use during Pregnancy and Offspring Conduct Problems: A Meta-Analysis. Neurosci. Biobehav. Rev..

[103] Tartaglione A.M., Venerosi A., Calamandrei G., Kostrzewa R.M., Archer T. (2015). Early-life toxic insults and onset of sporadic neurodegenerative diseases—An overview of experimental studies. Neurotoxin Modeling of Brain Disorders—Life-Long Outcomes in Behavioral Teratology.

[104] Wang X.-J., Xu W., Li J.-Q., Cao X.-P., Tan L., Yu J.-T. (2019). Early-Life Risk Factors for Dementia and Cognitive Impairment in Later Life: A Systematic Review and Meta-Analysis. J. Alzheimers Dis..

[105] Zuin M., Cervellati C., Brombo G., Trentini A., Roncon L., Zuliani G. (2021). Elevated Blood Homocysteine and Risk of Alzheimer’s Dementia: An Updated Systematic Review and Meta-Analysis Based on Prospective Studies. J. Prev. Alzheimers Dis..

[106] De Rooij S.R., Wouters H., Yonker J.E., Painter R.C., Roseboom T.J. (2010). Prenatal Undernutrition and Cognitive Function in Late Adulthood. Proc. Natl. Acad. Sci. USA.

[107] De Rooij S.R., Mutsaerts H.J.M.M., Petr J., Asllani I., Caan M.W.A., Groot P., Nederveen A.J., Schwab M., Roseboom T.J. (2019). Late-Life Brain Perfusion after Prenatal Famine Exposure. Neurobiol. Aging.

[108] Franke K., Gaser C., Roseboom T.J., Schwab M., De Rooij S.R. (2018). Premature Brain Aging in Humans Exposed to Maternal Nutrient Restriction during Early Gestation. NeuroImage.

[109] Wiegersma A.M., Boots A., Langendam M.W., Limpens J., Shenkin S.D., Korosi A., Roseboom T.J., De Rooij S.R. (2023). Do Prenatal Factors Shape the Risk for Dementia? A Systematic Review of the Epidemiological Evidence for the Prenatal Origins of Dementia. Soc. Psychiatry Psychiatr. Epidemiol..

[110] Boots A., Wiegersma A.M., Vali Y., Van Den Hof M., Langendam M.W., Limpens J., Backhouse E.V., Shenkin S.D., Wardlaw J.M., Roseboom T.J. (2023). Shaping the Risk for Late-Life Neurodegenerative Disease: A Systematic Review on Prenatal Risk Factors for Alzheimer’s Disease-Related Volumetric Brain Biomarkers. Neurosci. Biobehav. Rev..

[111] Gelfo F., Mandolesi L., Serra L., Sorrentino G., Caltagirone C. (2018). The Neuroprotective Effects of Experience on Cognitive Functions: Evidence from Animal Studies on the Neurobiological Bases of Brain Reserve. Neuroscience.

[112] Livingston G., Sommerlad A., Orgeta V., Costafreda S.G., Huntley J., Ames D., Ballard C., Banerjee S., Burns A., Cohen-Mansfield J. (2017). Dementia Prevention, Intervention, and Care. Lancet.

[113] Nithianantharajah J., Hannan A.J. (2011). Mechanisms Mediating Brain and Cognitive Reserve: Experience-Dependent Neuroprotection and Functional Compensation in Animal Models of Neurodegenerative Diseases. Prog. Neuropsychopharmacol. Biol. Psychiatry.

[114] Xu W., Tan L., Wang H.-F., Jiang T., Tan M.-S., Tan L., Zhao Q.-F., Li J.-Q., Wang J., Yu J.-T. (2015). Meta-Analysis of Modifiable Risk Factors for Alzheimer’s Disease. J. Neurol. Neurosurg. Psychiatry.

[115] Henderson A.S. (1988). The Risk Factors for Alzheimer’s Disease: A Review and a Hypothesis. Acta Psychiatr. Scand..

[116] Van Duijn C.M., Stijnen T., Hofman A., The Eurodem Risk Factors Research Group (1991). Risk Factors for Alzheimer’s Disease: Overview of the EURODEM Collaborative Re-Analysis of Case-Control Studies. Int. J. Epidemiol..

[117] Ballarini T., Melo Van Lent D., Brunner J., Schröder A., Wolfsgruber S., Altenstein S., Brosseron F., Buerger K., Dechent P., Dobisch L. (2021). Mediterranean Diet, Alzheimer Disease Biomarkers, and Brain Atrophy in Old Age. Neurology.

[118] Hachinski V., Einhäupl K., Ganten D., Alladi S., Brayne C., Stephan B.C.M., Sweeney M.D., Zlokovic B., Iturria-Medina Y., Iadecola C. (2019). Preventing Dementia by Preventing Stroke: The Berlin Manifesto. Alzheimers Dement..

[119] Lee C.M., Woodward M., Batty G.D., Beiser A.S., Bell S., Berr C., Bjertness E., Chalmers J., Clarke R., Dartigues J. (2020). Association of Anthropometry and Weight Change with Risk of Dementia and Its Major Subtypes: A Meta-analysis Consisting 2.8 Million Adults with 57 294 Cases of Dementia. Obes. Rev..

[120] Lee Y., Back J.H., Kim J., Kim S.-H., Na D.L., Cheong H.-K., Hong C.H., Kim Y.G. (2010). Systematic Review of Health Behavioral Risks and Cognitive Health in Older Adults. Int. Psychogeriatr..

[121] Plassman B.L. (2010). Systematic Review: Factors Associated with Risk for and Possible Prevention of Cognitive Decline in Later Life. Ann. Intern. Med..

[122] Seyedsalehi A., Warrier V., Bethlehem R.A.I., Perry B.I., Burgess S., Murray G.K. (2023). Educational Attainment, Structural Brain Reserve and Alzheimer’s Disease: A Mendelian Randomization Analysis. Brain.

[123] Schwarzinger M., Dufouil C. (2022). Forecasting the Prevalence of Dementia. Lancet Public Health.

[124] Litke R., Garcharna L.C., Jiwani S., Neugroschl J. (2021). Modifiable Risk Factors in Alzheimer Disease and Related Dementias: A Review. Clin. Ther..

[125] Pope S.K., Shue V.M., Beck C. (2003). Will a Healthy Lifestyle Help Prevent Alzheimer’s Disease?. Annu. Rev. Public Health.

[126] Haan M.N., Wallace R. (2004). Can Dementia Be Prevented? Brain Aging in a Population-Based Context. Annu. Rev. Public Health.

[127] Jansson E.T. (2005). Alzheimer Disease Is Substantially Preventable in the United States -- Review of Risk Factors, Therapy, and the Prospects for an Expert Software System. Med. Hypotheses.

[128] Middleton L., Yaffe K. (2009). Promising Strategies for the Prevention of Dementia. Arch. Neurol..

[129] Ritchie K., Carriere I., Ritchie C.W., Berr C., Artero S., Ancelin M.-L. (2010). Designing Prevention Programmes to Reduce Incidence of Dementia: Prospective Cohort Study of Modifiable Risk Factors. BMJ.

[130] Barnes D.E., Yaffe K. (2011). The Projected Impact of Risk Factor Reduction on Alzheimer’s Disease Prevalence. Lancet Neurol..

[131] Song X., Mitnitski A., Rockwood K. (2011). Nontraditional Risk Factors Combine to Predict Alzheimer Disease and Dementia. Neurology.

[132] Anstey K.J., Cherbuin N., Herath P.M. (2013). Development of a New Method for Assessing Global Risk of Alzheimer’s Disease for Use in Population Health Approaches to Prevention. Prev. Sci..

[133] Di Marco L.Y., Marzo A., Muñoz-Ruiz M., Ikram M.A., Kivipelto M., Ruefenacht D., Venneri A., Soininen H., Wanke I., Ventikos Y.A. (2014). Modifiable Lifestyle Factors in Dementia: A Systematic Review of Longitudinal Observational Cohort Studies. J. Alzheimers Dis..

[134] Anstey K.J., Eramudugolla R., Hosking D.E., Lautenschlager N.T., Dixon R.A. (2015). Bridging the Translation Gap: From Dementia Risk Assessment to Advice on Risk Reduction. J. Prev. Alzheimers Dis..

[135] Baumgart M., Snyder H.M., Carrillo M.C., Fazio S., Kim H., Johns H. (2015). Summary of the Evidence on Modifiable Risk Factors for Cognitive Decline and Dementia: A Population-based Perspective. Alzheimers Dement..

[136] Deckers K., Van Boxtel M.P.J., Schiepers O.J.G., De Vugt M., Muñoz Sánchez J.L., Anstey K.J., Brayne C., Dartigues J.-F., Engedal K., Kivipelto M. (2015). Target Risk Factors for Dementia Prevention: A Systematic Review and Delphi Consensus Study on the Evidence from Observational Studies: Major Risk Factors for Dementia Prevention. Int. J. Geriatr. Psychiatry.

[137] Hazar N., Seddigh L., Rampisheh Z., Nojomi M. (2016). Population Attributable Fraction of Modifiable Risk Factors for Alzheimer Disease: A Systematic Review of Systematic Reviews. Iran. J. Neurol..

[138] Killin L.O.J., Starr J.M., Shiue I.J., Russ T.C. (2016). Environmental Risk Factors for Dementia: A Systematic Review. BMC Geriatr..

[139] Wu Y.-T., Fratiglioni L., Matthews F.E., Lobo A., Breteler M.M.B., Skoog I., Brayne C. (2016). Dementia in Western Europe: Epidemiological Evidence and Implications for Policy Making. Lancet Neurol..

[140] Bellou V., Belbasis L., Tzoulaki I., Middleton L.T., Ioannidis J.P.A., Evangelou E. (2017). Systematic Evaluation of the Associations between Environmental Risk Factors and Dementia: An Umbrella Review of Systematic Reviews and Meta-Analyses. Alzheimers Dement..

[141] Rakesh G., Szabo S.T., Alexopoulos G.S., Zannas A.S. (2017). Strategies for Dementia Prevention: Latest Evidence and Implications. Ther. Adv. Chronic Dis..

[142] Larsson S.C., Markus H.S. (2018). Does Treating Vascular Risk Factors Prevent Dementia and Alzheimer’s Disease? A Systematic Review and Meta-Analysis. J. Alzheimers Dis..

[143] Anstey K.J., Ee N., Eramudugolla R., Jagger C., Peters R. (2019). A Systematic Review of Meta-Analyses That Evaluate Risk Factors for Dementia to Evaluate the Quantity, Quality, and Global Representativeness of Evidence. J. Alzheimers Dis..

[144] Armstrong R.A. (2019). Risk Factors for Alzheimer’s Disease. Folia Neuropathol..

[145] Edwards Iii G.A., Gamez N., Escobedo G., Calderon O., Moreno-Gonzalez I. (2019). Modifiable Risk Factors for Alzheimer’s Disease. Front. Aging Neurosci..

[146] Peters R., Booth A., Rockwood K., Peters J., D’Este C., Anstey K.J. (2019). Combining Modifiable Risk Factors and Risk of Dementia: A Systematic Review and Meta-Analysis. BMJ Open.

[147] Rochoy M., Bordet R., Gautier S., Chazard E. (2019). Factors Associated with the Onset of Alzheimer’s Disease: Data Mining in the French Nationwide Discharge Summary Database between 2008 and 2014. PLoS ONE.

[148] Yu J.-T., Xu W., Tan C.-C., Andrieu S., Suckling J., Evangelou E., Pan A., Zhang C., Jia J., Feng L. (2020). Evidence-Based Prevention of Alzheimer’s Disease: Systematic Review and Meta-Analysis of 243 Observational Prospective Studies and 153 Randomised Controlled Trials. J. Neurol. Neurosurg. Psychiatry.

[149] Liang J., Lu L., Li J., Qu X., Li J., Qian S., Wang Y., Jia R., Wang C., Xu Y. (2020). Contributions of Modifiable Risk Factors to Dementia Incidence: A Bayesian Network Analysis. J. Am. Med. Dir. Assoc..

[150] Kuo C.-Y., Stachiv I., Nikolai T. (2020). Association of Late Life Depression, Non-Modifiable Risk and Protective Factors with Dementia and Alzheimer’s Disease: Literature Review on Current Evidences, Preventive Interventions and Possible Future Trends in Prevention and Treatment of Dementia. Int. J. Environ. Res. Public. Health.

[151] Rolandi E., Zaccaria D., Vaccaro R., Abbondanza S., Pettinato L., Davin A., Guaita A. (2020). Estimating the Potential for Dementia Prevention through Modifiable Risk Factors Elimination in the Real-World Setting: A Population-Based Study. Alzheimers Res. Ther..

[152] Jones A., Ali M.U., Kenny M., Mayhew A., Mokashi V., He H., Lin S., Yavari E., Paik K., Subramanian D. (2024). Potentially Modifiable Risk Factors for Dementia and Mild Cognitive Impairment: An Umbrella Review and Meta-Analysis. Dement. Geriatr. Cogn. Disord..

[153] Stephan B.C.M., Cochrane L., Kafadar A.H., Brain J., Burton E., Myers B., Brayne C., Naheed A., Anstey K.J., Ashor A.W. (2024). Population Attributable Fractions of Modifiable Risk Factors for Dementia: A Systematic Review and Meta-Analysis. Lancet Healthy Longev..

[154] Larsson S.C., Traylor M., Malik R., Dichgans M., Burgess S., Markus H.S. (2017). Modifiable Pathways in Alzheimer’s Disease: Mendelian Randomisation Analysis. BMJ.

[155] Norton S., Matthews F.E., Barnes D.E., Yaffe K., Brayne C. (2014). Potential for Primary Prevention of Alzheimer’s Disease: An Analysis of Population-Based Data. Lancet Neurol..

[156] Contador I., Bermejo-Pareja F., Puertas-Martin V., Benito-Leon J. (2015). Childhood and Adulthood Rural Residence Increases the Risk of Dementia: NEDICES Study. Curr. Alzheimer Res..

[157] Dekhtyar S., Wang H.-X., Fratiglioni L., Herlitz A. (2016). Childhood School Performance, Education and Occupational Complexity: A Life-Course Study of Dementia in the Kungsholmen Project. Int. J. Epidemiol..

[158] Scarmeas N., Stern Y. (2004). Cognitive Reserve: Implications for Diagnosis and Prevention of Alzheimer’s Disease. Curr. Neurol. Neurosci. Rep..

[159] Stern Y. (2006). Cognitive Reserve and Alzheimer Disease. Alzheimer Dis. Assoc. Disord..

[160] Saraulli D., Costanzi M., Mastrorilli V., Farioli-Vecchioli S. (2017). The Long Run: Neuroprotective Effects of Physical Exercise on Adult Neurogenesis from Youth to Old Age. Curr. Neuropharmacol..

[161] Steiner B., Wolf S.A., Kempermann G. (2006). Adult Neurogenesis and Neurodegenerative Disease. Regen. Med..

[162] Xu W., Yu J.-T., Tan M.-S., Tan L. (2015). Cognitive Reserve and Alzheimer’s Disease. Mol. Neurobiol..

[163] He C., Tsipis C.P., LaManna J.C., Xu K., Halpern H.J., LaManna J.C., Harrison D.K., Epel B. (2017). Environmental enrichment induces increased cerebral capillary density and improved cognitive function in mice. Oxygen Transport to Tissue XXXIX.

[164] Ramos-Miguel A., Jones A.A., Sawada K., Barr A.M., Bayer T.A., Falkai P., Leurgans S.E., Schneider J.A., Bennett D.A., Honer W.G. (2018). Frontotemporal Dysregulation of the SNARE Protein Interactome Is Associated with Faster Cognitive Decline in Old Age. Neurobiol. Dis..

[165] Walker C.K., Herskowitz J.H. (2021). Dendritic Spines: Mediators of Cognitive Resilience in Aging and Alzheimer’s Disease. Neuroscience.

[166] Arenaza-Urquijo E.M., Landeau B., La Joie R., Mevel K., Mézenge F., Perrotin A., Desgranges B., Bartrés-Faz D., Eustache F., Chételat G. (2013). Relationships between Years of Education and Gray Matter Volume, Metabolism and Functional Connectivity in Healthy Elders. NeuroImage.

[167] Bozzali M., Dowling C., Serra L., Spanò B., Torso M., Marra C., Castelli D., Dowell N.G., Koch G., Caltagirone C. (2015). The Impact of Cognitive Reserve on Brain Functional Connectivity in Alzheimer’s Disease. J. Alzheimers Dis..

[168] Perani D., Farsad M., Ballarini T., Lubian F., Malpetti M., Fracchetti A., Magnani G., March A., Abutalebi J. (2017). The Impact of Bilingualism on Brain Reserve and Metabolic Connectivity in Alzheimer’s Dementia. Proc. Natl. Acad. Sci. USA.

[169] Van Balkom T.D., Van Den Heuvel O.A., Berendse H.W., Van Der Werf Y.D., Vriend C. (2020). The Effects of Cognitive Training on Brain Network Activity and Connectivity in Aging and Neurodegenerative Diseases: A Systematic Review. Neuropsychol. Rev..

[170] Varela-López B., Cruz-Gómez Á.J., Lojo-Seoane C., Díaz F., Pereiro A.X., Zurrón M., Lindín M., Galdo-Álvarez S. (2022). Cognitive Reserve, Neurocognitive Performance, and High-Order Resting-State Networks in Cognitively Unimpaired Aging. Neurobiol. Aging.

[171] Adesuyan M., Jani Y.H., Alsugeir D., Howard R., Wong I.C.K., Wei L., Brauer R. (2023). Trends in the Incidence of Dementia in People with Hypertension in the UK 2000 to 2021. Alzheimers Dement. Diagn. Assess. Dis. Monit..

[172] Lennon M.J., Makkar S.R., Crawford J.D., Sachdev P.S. (2019). Midlife Hypertension and Alzheimer’s Disease: A Systematic Review and Meta-Analysis. J. Alzheimers Dis..

[173] Power M.C., Weuve J., Gagne J.J., McQueen M.B., Viswanathan A., Blacker D. (2011). The Association Between Blood Pressure and Incident Alzheimer Disease: A Systematic Review and Meta-Analysis. Epidemiology.

[174] Rouch L., Cestac P., Hanon O., Cool C., Helmer C., Bouhanick B., Chamontin B., Dartigues J.-F., Vellas B., Andrieu S. (2015). Antihypertensive Drugs, Prevention of Cognitive Decline and Dementia: A Systematic Review of Observational Studies, Randomized Controlled Trials and Meta-Analyses, with Discussion of Potential Mechanisms. CNS Drugs.

[175] Daugherty A.M. (2021). Hypertension-Related Risk for Dementia: A Summary Review with Future Directions. Semin. Cell Dev. Biol..

[176] Hughes T.M., Sink K.M. (2016). Hypertension and Its Role in Cognitive Function: Current Evidence and Challenges for the Future. Am. J. Hypertens..

[177] Liu Y., Zhong X., Shen J., Jiao L., Tong J., Zhao W., Du K., Gong S., Liu M., Wei M. (2020). Elevated Serum TC and LDL-C Levels in Alzheimer’s Disease and Mild Cognitive Impairment: A Meta-Analysis Study. Brain Res..

[178] Espeland M.A., Newman A.B., Sink K., Gill T.M., King A.C., Miller M.E., Guralnik J., Katula J., Church T., Manini T. (2015). Associations Between Ankle-Brachial Index and Cognitive Function: Results from the Lifestyle Interventions and Independence for Elders Trial. J. Am. Med. Dir. Assoc..

[179] Guerchet M., Aboyans V., Nubukpo P., Lacroix P., Clément J.-P., Preux P.-M. (2011). Ankle-Brachial Index as a Marker of Cognitive Impairment and Dementia in General Population. A Systematic Review. Atherosclerosis.

[180] Anstey K.J., Cherbuin N., Budge M., Young J. (2011). Body Mass Index in Midlife and Late-Life as a Risk Factor for Dementia: A Meta-Analysis of Prospective Studies: BMI and Risk of Dementia. Obes. Rev..

[181] Zhou F., Chen S. (2019). Hyperhomocysteinemia and Risk of Incident Cognitive Outcomes: An Updated Dose-Response Meta-Analysis of Prospective Cohort Studies. Ageing Res. Rev..

[182] Kunutsor S.K., Isiozor N.M., Voutilainen A., Laukkanen J.A. (2022). Handgrip Strength and Risk of Cognitive Outcomes: New Prospective Study and Meta-Analysis of 16 Observational Cohort Studies. GeroScience.

[183] Zuin M., Roncon L., Passaro A., Cervellati C., Zuliani G. (2021). Metabolic Syndrome and the Risk of Late Onset Alzheimer’s Disease: An Updated Review and Meta-Analysis. Nutr. Metab. Cardiovasc. Dis..

[184] Sabia S., Fayosse A., Dumurgier J., Schnitzler A., Empana J.-P., Ebmeier K.P., Dugravot A., Kivimäki M., Singh-Manoux A. (2019). Association of Ideal Cardiovascular Health at Age 50 with Incidence of Dementia: 25 Year Follow-up of Whitehall II Cohort Study. BMJ.

[185] Kivimäki M., Singh-Manoux A., Pentti J., Sabia S., Nyberg S.T., Alfredsson L., Goldberg M., Knutsson A., Koskenvuo M., Koskinen A. (2019). Physical Inactivity, Cardiometabolic Disease, and Risk of Dementia: An Individual-Participant Meta-Analysis. BMJ.

[186] Rahmani J., Roudsari A.H., Bawadi H., Clark C., Ryan P.M., Salehisahlabadi A., Rahimi Sakak F., Goodarzi N., Razaz J.M. (2022). Body Mass Index and Risk of Parkinson, Alzheimer, Dementia, and Dementia Mortality: A Systematic Review and Dose–Response Meta-Analysis of Cohort Studies among 5 Million Participants. Nutr. Neurosci..

[187] Wang C., Fu W., Cao S., Jiang H., Guo Y., Xv H., Liu J., Gan Y., Lu Z. (2021). Weight Loss and the Risk of Dementia: A Meta-Analysis of Cohort Studies. Curr. Alzheimer Res..

[188] Vagelatos N.T., Eslick G.D. (2013). Type 2 Diabetes as a Risk Factor for Alzheimer’s Disease: The Confounders, Interactions, and Neuropathology Associated with This Relationship. Epidemiol. Rev..

[189] Debette S., Seshadri S., Beiser A., Au R., Himali J.J., Palumbo C., Wolf P.A., DeCarli C. (2011). Midlife Vascular Risk Factor Exposure Accelerates Structural Brain Aging and Cognitive Decline. Neurology.

[190] Cox S.R., Lyall D.M., Ritchie S.J., Bastin M.E., Harris M.A., Buchanan C.R., Fawns-Ritchie C., Barbu M.C., De Nooij L., Reus L.M. (2019). Associations between Vascular Risk Factors and Brain MRI Indices in UK Biobank. Eur. Heart J..

[191] Azarpazhooh M.R., Avan A., Cipriano L.E., Munoz D.G., Sposato L.A., Hachinski V. (2018). Concomitant Vascular and Neurodegenerative Pathologies Double the Risk of Dementia. Alzheimers Dement..

[192] Bilgel M., Bannerjee A., Shafer A., An Y., Resnick S.M. (2021). Vascular Risk Is Not Associated with PET Measures of Alzheimer’s Disease Neuropathology among Cognitively Normal Older Adults. Neuroimage Rep..

[193] You Y., Liu Z., Chen Y., Xu Y., Qin J., Guo S., Huang J., Tao J. (2021). The Prevalence of Mild Cognitive Impairment in Type 2 Diabetes Mellitus Patients: A Systematic Review and Meta-Analysis. Acta Diabetol..

[194] Kopf D., Frölich L. (2009). Risk of Incident Alzheimer’s Disease in Diabetic Patients: A Systematic Review of Prospective Trials. J. Alzheimers Dis..

[195] Profenno L.A., Porsteinsson A.P., Faraone S.V. (2010). Meta-Analysis of Alzheimer’s Disease Risk with Obesity, Diabetes, and Related Disorders. Biol. Psychiatry.

[196] Zhang J., Chen C., Hua S., Liao H., Wang M., Xiong Y., Cao F. (2017). An Updated Meta-Analysis of Cohort Studies: Diabetes and Risk of Alzheimer’s Disease. Diabetes Res. Clin. Pract..

[197] Cooper C., Sommerlad A., Lyketsos C.G., Livingston G. (2015). Modifiable Predictors of Dementia in Mild Cognitive Impairment: A Systematic Review and Meta-Analysis. Am. J. Psychiatry.

[198] Li L., Cavuoto M., Biddiscombe K., Pike K.E. (2020). Diabetes Mellitus Increases Risk of Incident Dementia in APOE ε4 Carriers: A Meta-Analysis. J. Alzheimers Dis..

[199] Loughrey D.G., Kelly M.E., Kelley G.A., Brennan S., Lawlor B.A. (2018). Association of Age-Related Hearing Loss with Cognitive Function, Cognitive Impairment, and Dementia: A Systematic Review and Meta-Analysis. JAMA Otolaryngol. Neck Surg..

[200] Morita Y., Sasaki T., Takahashi K., Kitazawa M., Nonomura Y., Yagi C., Yamagishi T., Ohshima S., Izumi S., Wakasugi M. (2019). Age-Related Hearing Loss Is Strongly Associated with Cognitive Decline Regardless of the APOE4 Polymorphism. Otol. Neurotol..

[201] Griffiths T.D., Lad M., Kumar S., Holmes E., McMurray B., Maguire E.A., Billig A.J., Sedley W. (2020). How Can Hearing Loss Cause Dementia?. Neuron.

[202] Armstrong N.M., An Y., Doshi J., Erus G., Ferrucci L., Davatzikos C., Deal J.A., Lin F.R., Resnick S.M. (2019). Association of Midlife Hearing Impairment with Late-Life Temporal Lobe Volume Loss. JAMA Otolaryngol. Neck Surg..

[203] Fortunato S., Forli F., Guglielmi V., De Corso E., Paludetti G., Berrettini S., FetonI A.R. (2016). Ipoacusia e Declino Cognitivo: Revisione Della Letteratura. Acta Otorhinolaryngol. ital..

[204] Uchida Y., Sugiura S., Nishita Y., Saji N., Sone M., Ueda H. (2019). Age-Related Hearing Loss and Cognitive Decline—The Potential Mechanisms Linking the Two. Auris. Nasus. Larynx.

[205] Fleminger S. (2003). Head Injury as a Risk Factor for Alzheimer’s Disease: The Evidence 10 Years on; a Partial Replication. J. Neurol. Neurosurg. Psychiatry.

[206] Gu D., Ou S., Liu G. (2022). Traumatic Brain Injury and Risk of Dementia and Alzheimer’s Disease: A Systematic Review and Meta-Analysis. Neuroepidemiology.

[207] Mendez M.F. (2017). What Is the Relationship of Traumatic Brain Injury to Dementia?. J. Alzheimers Dis..

[208] Perry D.C., Sturm V.E., Peterson M.J., Pieper C.F., Bullock T., Boeve B.F., Miller B.L., Guskiewicz K.M., Berger M.S., Kramer J.H. (2016). Association of Traumatic Brain Injury with Subsequent Neurological and Psychiatric Disease: A Meta-Analysis. J. Neurosurg..

[209] Crane P.K., Gibbons L.E., Dams-O’Connor K., Trittschuh E., Leverenz J.B., Keene C.D., Sonnen J., Montine T.J., Bennett D.A., Leurgans S. (2016). Association of Traumatic Brain Injury with Late-Life Neurodegenerative Conditions and Neuropathologic Findings. JAMA Neurol..

[210] Cechetto D.F., Hachinski V., Whitehead S.N. (2008). Vascular Risk Factors and Alzheimer’s Disease. Expert Rev. Neurother..

[211] Pinho J., Quintas-Neves M., Dogan I., Reetz K., Reich A., Costa A.S. (2021). Incident Stroke in Patients with Alzheimer’s Disease: Systematic Review and Meta-Analysis. Sci. Rep..

[212] Barba R., Martínez-Espinosa S., Rodríguez-García E., Pondal M., Vivancos J., Del Ser T. (2000). Poststroke Dementia: Clinical Features and Risk Factors. Stroke.

[213] Barba R., Castro M.D., Del Mar Morín M., Rodriguez-Romero R., Rodríguez-García E., Cantón R., Del Ser T. (2001). Prestroke Dementia. Cerebrovasc. Dis..

[214] Jiang W., Liang G.-H., Li J.-A., Yu P., Dong M. (2022). Migraine and the Risk of Dementia: A Meta-Analysis and Systematic Review. Aging Clin. Exp. Res..

[215] Huang L., Fu C., Li J., Peng S. (2022). Late-Onset Epilepsy and the Risk of Dementia: A Systematic Review and Meta-Analysis. Aging Clin. Exp. Res..

[216] Zhao N., Chen H., Zhang W., Yao J., Tu Q., Yu X., Sun X. (2022). Bidirectional Influences between Seizures and Dementia: A Systematic Review and Meta-analysis. Int. J. Geriatr. Psychiatry.

[217] Altuna M., Olmedo-Saura G., Carmona-Iragui M., Fortea J. (2022). Mechanisms Involved in Epileptogenesis in Alzheimer’s Disease and Their Therapeutic Implications. Int. J. Mol. Sci..

[218] Diniz B.S., Butters M.A., Albert S.M., Dew M.A., Reynolds C.F. (2013). Late-Life Depression and Risk of Vascular Dementia and Alzheimer’s Disease: Systematic Review and Meta-Analysis of Community-Based Cohort Studies. Br. J. Psychiatry.

[219] Mehta K., Thandavan S.P., Mohebbi M., Pasco J.A., Williams L.J., Walder K., Ng B.L., Gupta V.B. (2022). Depression and Bone Loss as Risk Factors for Cognitive Decline: A Systematic Review and Meta-Analysis. Ageing Res. Rev..

[220] Santabárbara Serrano J., Sevil Pérez A., Olaya B., Gracia García P., López Antón R. (2019). Depresión tardía clínicamente relevante y riesgo de demencia: Revisión sistemática y metaanálisis de estudios prospectivos de cohortes. Rev. Neurol..

[221] Williams L.M. (2016). Precision Psychiatry: A Neural Circuit Taxonomy for Depression and Anxiety. Lancet Psychiatry.

[222] Harrington K.D., Lim Y.Y., Gould E., Maruff P. (2015). Amyloid-Beta and Depression in Healthy Older Adults: A Systematic Review. Aust. N. Z. J. Psychiatry.

[223] Cai W.-J., Tian Y., Ma Y.-H., Dong Q., Tan L., Yu J.-T., Alzheimer’s Disease Neuroimaging Initiative (2021). Associations of Anxiety with Amyloid, Tau, and Neurodegeneration in Older Adults without Dementia: A Longitudinal Study. J. Alzheimers Dis..

[224] Hanseeuw B.J., Jonas V., Jackson J., Betensky R.A., Rentz D.M., Johnson K.A., Sperling R.A., Donovan N.J. (2020). Association of Anxiety with Subcortical Amyloidosis in Cognitively Normal Older Adults. Mol. Psychiatry.

[225] Becker E., Orellana Rios C.L., Lahmann C., Rücker G., Bauer J., Boeker M. (2018). Anxiety as a Risk Factor of Alzheimer’s Disease and Vascular Dementia. Br. J. Psychiatry.

[226] Luo J., Beam C.R., Gatz M. (2023). Is Stress an Overlooked Risk Factor for Dementia? A Systematic Review from a Lifespan Developmental Perspective. Prev. Sci..

[227] Bubu O.M., Brannick M., Mortimer J., Umasabor-Bubu O., Sebastião Y.V., Wen Y., Schwartz S., Borenstein A.R., Wu Y., Morgan D. (2017). Sleep, Cognitive Impairment, and Alzheimer’s Disease: A Systematic Review and Meta-Analysis. Sleep.

[228] Wu L., Sun D., Tan Y. (2018). A Systematic Review and Dose-Response Meta-Analysis of Sleep Duration and the Occurrence of Cognitive Disorders. Sleep Breath..

[229] Sabia S., Fayosse A., Dumurgier J., Van Hees V.T., Paquet C., Sommerlad A., Kivimäki M., Dugravot A., Singh-Manoux A. (2021). Association of Sleep Duration in Middle and Old Age with Incidence of Dementia. Nat. Commun..

[230] López-García S., Lage C., Pozueta A., García-Martínez M., Kazimierczak M., Fernández-Rodríguez A., Bravo M., Reyes-González L., Irure J., López-Hoyos M. (2021). Sleep Time Estimated by an Actigraphy Watch Correlates with CSF Tau in Cognitively Unimpaired Elders: The Modulatory Role of APOE. Front. Aging Neurosci..

[231] Aini N., Chu H., Banda K.J., Chen R., Lee T.-Y., Pien L.-C., Liu D., Lai Y.-J., Kang X.L., Chou K.-R. (2023). Prevalence of Sleep-Related Breathing Disorders and Associated Risk Factors among People with Dementia: A Meta-Analysis. Sleep Med..

[232] Shi L., Chen S.-J., Ma M.-Y., Bao Y.-P., Han Y., Wang Y.-M., Shi J., Vitiello M.V., Lu L. (2018). Sleep Disturbances Increase the Risk of Dementia: A Systematic Review and Meta-Analysis. Sleep Med. Rev..

[233] Snyder B., Shell B., Cunningham J.T., Cunningham R.L. (2017). Chronic Intermittent Hypoxia Induces Oxidative Stress and Inflammation in Brain Regions Associated with Early-Stage Neurodegeneration. Physiol. Rep..

[234] Canevelli M., Wallace L.M.K., Bruno G., Cesari M., Rockwood K., Ward D.D. (2024). Frailty Is Associated with the Clinical Expression of Neuropsychological Deficits in Older Adults. Eur. J. Neurol..

[235] García-Chanes R.E., Avila-Funes J.A., Borda M.G., Pérez-Zepeda M.U., Gutiérrez-Robledo L.M. (2023). Higher Frailty Levels Are Associated with Lower Cognitive Test Scores in a Multi-Country Study: Evidence from the Study on Global Ageing and Adult Health. Front. Med..

[236] Bermejo-Pareja F., Gómez De La Cámara A., Del Ser T., Contador I., Llamas-Velasco S., López-Arrieta J.M., Martín-Arriscado C., Hernández-Gallego J., Vega S., Benito-León J. (2022). The Health Status: The Ignored Risk Factor in Dementia Incidence. NEDICES Cohort. Aging Clin. Exp. Res..

[237] Sargent L., Nalls M., Amella E.J., Slattum P.W., Mueller M., Bandinelli S., Tian Q., Swift-Scanlan T., Lageman S.K., Singleton A. (2020). Shared Mechanisms for Cognitive Impairment and Physical Frailty: A Model for Complex Systems. Alzheimers Dement. Transl. Res. Clin. Interv..

[238] Wallace L.M.K., Theou O., Godin J., Andrew M.K., Bennett D.A., Rockwood K. (2019). Investigation of Frailty as a Moderator of the Relationship between Neuropathology and Dementia in Alzheimer’s Disease: A Cross-Sectional Analysis of Data from the Rush Memory and Aging Project. Lancet Neurol..

[239] Langballe E.M., Ask H., Holmen J., Stordal E., Saltvedt I., Selbæk G., Fikseaunet A., Bergh S., Nafstad P., Tambs K. (2015). Alcohol Consumption and Risk of Dementia up to 27 Years Later in a Large, Population-Based Sample: The HUNT Study, Norway. Eur. J. Epidemiol..

[240] Wang G., Li D.Y., Vance D.E., Li W. (2023). Alcohol Use Disorder as a Risk Factor for Cognitive Impairment. J. Alzheimers Dis..

[241] Rehm J., Hasan O.S.M., Black S.E., Shield K.D., Schwarzinger M. (2019). Alcohol Use and Dementia: A Systematic Scoping Review. Alzheimers Res. Ther..

[242] Xu W., Wang H., Wan Y., Tan C., Li J., Tan L., Yu J.-T. (2017). Alcohol Consumption and Dementia Risk: A Dose–Response Meta-Analysis of Prospective Studies. Eur. J. Epidemiol..

[243] Ganguli M., Bilt J.V., Saxton J.A., Shen C., Dodge H.H. (2005). Alcohol Consumption and Cognitive Function in Late Life: A Longitudinal Community Study. Neurology.

[244] Ruitenberg A., Van Swieten J.C., Witteman J.C., Mehta K.M., Van Duijn C.M., Hofman A., Breteler M.M. (2002). Alcohol Consumption and Risk of Dementia: The Rotterdam Study. Lancet.

[245] Xie C., Feng Y. (2022). Alcohol Consumption and Risk of Alzheimer’s Disease: A Dose–Response Meta-analysis. Geriatr. Gerontol. Int..

[246] Overview—Dementia, Disability and Frailty in Later Life—Mid-Life Approaches to Delay or Prevent Onset—Guidance—NICE. https://www.nice.org.uk/guidance/ng16.

[247] Niu H., Qu Y., Li Z., Wang R., Li L., Li M., Lv X., Gao C., Song Y., Li B. (2018). Smoking and Risk for Alzheimer Disease: A Meta-Analysis Based on Both Case-Control and Cohort Study. J. Nerv. Ment. Dis..

[248] Zhong G., Wang Y., Zhang Y., Guo J.J., Zhao Y. (2015). Smoking Is Associated with an Increased Risk of Dementia: A Meta-Analysis of Prospective Cohort Studies with Investigation of Potential Effect Modifiers. PLoS ONE.

[249] Liu Y., Li H., Wang J., Xue Q., Yang X., Kang Y., Li M., Xu J., Li G., Li C. (2020). Association of Cigarette Smoking with Cerebrospinal Fluid Biomarkers of Neurodegeneration, Neuroinflammation, and Oxidation. JAMA Netw. Open.

[250] Ruan Y., Tang J., Guo X., Li K., Li D. (2018). Dietary Fat Intake and Risk of Alzheimer’s Disease and Dementia: A Meta-Analysis of Cohort Studies. Curr. Alzheimer Res..

[251] Cabrera C., Vicens P., Torrente M. (2021). Modifiable Risk Factors for Dementia: The Role of Gut Microbiota. Curr. Alzheimer Res..

[252] Aridi Y., Walker J., Wright O. (2017). The Association between the Mediterranean Dietary Pattern and Cognitive Health: A Systematic Review. Nutrients.

[253] Samadi M., Moradi S., Moradinazar M., Mostafai R., Pasdar Y. (2019). Dietary Pattern in Relation to the Risk of Alzheimer’s Disease: A Systematic Review. Neurol. Sci..

[254] Talebi S., Asoudeh F., Naeini F., Sadeghi E., Travica N., Mohammadi H. (2023). Association between Animal Protein Sources and Risk of Neurodegenerative Diseases: A Systematic Review and Dose-Response Meta-Analysis. Nutr. Rev..

[255] Solfrizzi V., Custodero C., Lozupone M., Imbimbo B.P., Valiani V., Agosti P., Schilardi A., D’Introno A., La Montagna M., Calvani M. (2017). Relationships of Dietary Patterns, Foods, and Micro- and Macronutrients with Alzheimer’s Disease and Late-Life Cognitive Disorders: A Systematic Review. J. Alzheimers Dis..

[256] Fotuhi M., Mohassel P., Yaffe K. (2009). Fish Consumption, Long-Chain Omega-3 Fatty Acids and Risk of Cognitive Decline or Alzheimer Disease: A Complex Association. Nat. Rev. Neurol..

[257] Li F.-J., Shen L., Ji H.-F. (2012). Dietary Intakes of Vitamin E, Vitamin C, and β-Carotene and Risk of Alzheimer’s Disease: A Meta-Analysis. J. Alzheimers Dis..

[258] Littlejohns T.J., Henley W.E., Lang I.A., Annweiler C., Beauchet O., Chaves P.H.M., Fried L., Kestenbaum B.R., Kuller L.H., Langa K.M. (2014). Vitamin D and the Risk of Dementia and Alzheimer Disease. Neurology.

[259] Kalra A., Teixeira A.L., Diniz B.S. (2019). Association of Vitamin D Levels with Incident All Cause Dementia in Longitudinal Observational Studies: A Systematic Review and Meta-Analysis. J. Prev. Alzheimers Dis..

[260] Chai B., Gao F., Wu R., Dong T., Gu C., Lin Q., Zhang Y. (2019). Vitamin D Deficiency as a Risk Factor for Dementia and Alzheimer’s Disease: An Updated Meta-Analysis. BMC Neurol..

[261] Jayedi A., Rashidy-Pour A., Shab-Bidar S. (2019). Vitamin D Status and Risk of Dementia and Alzheimer’s Disease: A Meta-Analysis of Dose-Response. Nutr. Neurosci..

[262] Shen L., Ji H.-F. (2015). Vitamin D Deficiency Is Associated with Increased Risk of Alzheimer’s Disease and Dementia: Evidence from Meta-Analysis. Nutr. J..

[263] Hersi M., Irvine B., Gupta P., Gomes J., Birkett N., Krewski D. (2017). Risk Factors Associated with the Onset and Progression of Alzheimer’s Disease: A Systematic Review of the Evidence. NeuroToxicology.

[264] Hörder H., Johansson L., Guo X., Grimby G., Kern S., Östling S., Skoog I. (2018). Midlife Cardiovascular Fitness and Dementia: A 44-Year Longitudinal Population Study in Women. Neurology.

[265] Spartano N.L., Ngandu T. (2018). Fitness and Dementia Risk: Further Evidence of the Heart-Brain Connection. Neurology.

[266] Collins A.M., Molina-Hidalgo C., Aghjayan S.L., Fanning J., Erlenbach E.D., Gothe N.P., Velazquez-Diaz D., Erickson K.I. (2023). Differentiating the Influence of Sedentary Behavior and Physical Activity on Brain Health in Late Adulthood. Exp. Gerontol..

[267] Dorsman K.A., Weiner-Light S., Staffaroni A.M., Brown J.A., Wolf A., Cobigo Y., Walters S., Kramer J.H., Casaletto K.B. (2020). Get Moving! Increases in Physical Activity Are Associated with Increasing Functional Connectivity Trajectories in Typically Aging Adults. Front. Aging Neurosci..

[268] Erickson K.I., Donofry S.D., Sewell K.R., Brown B.M., Stillman C.M. (2022). Cognitive Aging and the Promise of Physical Activity. Annu. Rev. Clin. Psychol..

[269] Huang A.R., Roth D.L., Cidav T., Chung S., Amjad H., Thorpe R.J., Boyd C.M., Cudjoe T.K.M. (2023). Social Isolation and 9-year Dementia Risk in community-dwelling Medicare Beneficiaries in the United States. J. Am. Geriatr. Soc..

[270] Evans I.E.M., Martyr A., Collins R., Brayne C., Clare L. (2019). Social Isolation and Cognitive Function in Later Life: A Systematic Review and Meta-Analysis. J. Alzheimers Dis..

[271] Najar J., Aakre J.A., Vassilaki M., Wetterberg H., Rydén L., Zettergren A., Skoog I., Jack C.R., Knopman D.S., Petersen R.C. (2021). Sex Difference in the Relation Between Marital Status and Dementia Risk in Two Population-Based Cohorts. J. Alzheimers Dis..

[272] Biddle K.D., Jacobs H.I.L., D’Oleire Uquillas F., Zide B.S., Kirn D.R., Properzi M.R., Rentz D.M., Johnson K.A., Sperling R.A., Donovan N.J. (2020). Associations of Widowhood and β-Amyloid with Cognitive Decline in Cognitively Unimpaired Older Adults. JAMA Netw. Open.

[273] Sundström A., Westerlund O., Mousavi-Nasab H., Adolfsson R., Nilsson L.G. (2014). The Relationship between Marital and Parental Status and the Risk of Dementia. Int. Psychogeriatr..

[274] Guarnera J., Yuen E., Macpherson H. (2023). The Impact of Loneliness and Social Isolation on Cognitive Aging: A Narrative Review. J. Alzheimers Dis. Rep..

[275] Lam J.A., Murray E.R., Yu K.E., Ramsey M., Nguyen T.T., Mishra J., Martis B., Thomas M.L., Lee E.E. (2021). Neurobiology of Loneliness: A Systematic Review. Neuropsychopharmacology.

[276] Calderón-Garcidueñas L., González-Maciel A., Kulesza R.J., González-González L.O., Reynoso-Robles R., Mukherjee P.S., Torres-Jardón R. (2019). Air Pollution, Combustion and Friction Derived Nanoparticles, and Alzheimer’s Disease in Urban Children and Young Adults. J. Alzheimers Dis..

[277] Carey I.M., Anderson H.R., Atkinson R.W., Beevers S.D., Cook D.G., Strachan D.P., Dajnak D., Gulliver J., Kelly F.J. (2018). Are Noise and Air Pollution Related to the Incidence of Dementia? A Cohort Study in London, England. BMJ Open.

[278] Jung C.-R., Lin Y.-T., Hwang B.-F. (2015). Ozone, Particulate Matter, and Newly Diagnosed Alzheimer’s Disease: A Population-Based Cohort Study in Taiwan. J. Alzheimers Dis..

[279] Yuan S., Huang X., Zhang L., Ling Y., Tan S., Peng M., Xu A., Lyu J. (2023). Associations of Air Pollution with All-Cause Dementia, Alzheimer’s Disease, and Vascular Dementia: A Prospective Cohort Study Based on 437,932 Participants from the UK Biobank. Front. Neurosci..

[280] Zhang B., Weuve J., Langa K.M., D’Souza J., Szpiro A., Faul J., Mendes De Leon C., Gao J., Kaufman J.D., Sheppard L. (2023). Comparison of Particulate Air Pollution from Different Emission Sources and Incident Dementia in the US. JAMA Intern. Med..

[281] Peters R., Ee N., Peters J., Booth A., Mudway I., Anstey K.J. (2019). Air Pollution and Dementia: A Systematic Review. J. Alzheimers Dis..

[282] Hussenoeder F.S., Riedel-Heller S.G. (2018). Primary Prevention of Dementia: From Modifiable Risk Factors to a Public Brain Health Agenda?. Soc. Psychiatry Psychiatr. Epidemiol..

[283] Rosenberg A., Ngandu T., Rusanen M., Antikainen R., Bäckman L., Havulinna S., Hänninen T., Laatikainen T., Lehtisalo J., Levälahti E. (2018). Multidomain Lifestyle Intervention Benefits a Large Elderly Population at Risk for Cognitive Decline and Dementia Regardless of Baseline Characteristics: The FINGER Trial. Alzheimers Dement..

[284] Andrieu S., Guyonnet S., Coley N., Cantet C., Bonnefoy M., Bordes S., Bories L., Cufi M.-N., Dantoine T., Dartigues J.-F. (2017). Effect of Long-Term Omega 3 Polyunsaturated Fatty Acid Supplementation with or without Multidomain Intervention on Cognitive Function in Elderly Adults with Memory Complaints (MAPT): A Randomised, Placebo-Controlled Trial. Lancet Neurol..

[285] Van Charante E.P.M., Richard E., Eurelings L.S., Van Dalen J.-W., Ligthart S.A., Van Bussel E.F., Hoevenaar-Blom M.P., Vermeulen M., Van Gool W.A. (2016). Effectiveness of a 6-Year Multidomain Vascular Care Intervention to Prevent Dementia (preDIVA): A Cluster-Randomised Controlled Trial. Lancet.

[286] Takada L.T., Aláez-Verson C., Burgute B.D., Nitrini R., Sosa A.L., Castilhos R.M., Chaves M.F., Longoria E.-M., Carrillo-Sánchez K., Brucki S.M.D. (2022). Discovery and Validation of Dominantly Inherited Alzheimer’s Disease Mutations in Populations from Latin America. Alzheimers Res. Ther..

[287] Lai D., Zhang M., Li R., Zhang C., Zhang P., Liu Y., Gao S., Foroud T. (2023). Identifying Genes Associated with Alzheimer’s Disease Using Gene-Based Polygenic Risk Score. J. Alzheimers Dis..

[288] Maloney B., Lahiri D.K. (2016). Epigenetics of Dementia: Understanding the Disease as a Transformation Rather than a State. Lancet Neurol..

[289] Bennett D.A., Buchman A.S., Boyle P.A., Barnes L.L., Wilson R.S., Schneider J.A. (2018). Religious Orders Study and Rush Memory and Aging Project. J. Alzheimers Dis..

[290] Kunkle B.W., Grenier-Boley B., Sims R., Bis J.C., Damotte V., Naj A.C., Alzheimer Disease Genetics Consortium (ADGC), The European Alzheimer’s Disease Initiative (EADI), Cohorts for Heart and Aging Research in Genomic Epidemiology Consortium (CHARGE), Genetic and Environmental Risk in AD/Defining Genetic, Polygenic and Environmental Risk for Alzheimer’s Disease Consortium (GERAD/PERADES) (2019). Genetic Meta-Analysis of Diagnosed Alzheimer’s Disease Identifies New Risk Loci and Implicates Aβ, Tau, Immunity and Lipid Processing. Nat. Genet..

[291] Knopman D.S., Roberts R. (2010). Vascular Risk Factors: Imaging and Neuropathologic Correlates. J. Alzheimers Dis..

[292] Azarpazhooh M.R., Avan A., Cipriano L.E., Munoz D.G., Erfanian M., Amiri A., Stranges S., Hachinski V. (2020). A Third of Community-Dwelling Elderly with Intermediate and High Level of Alzheimer’s Neuropathologic Changes Are Not Demented: A Meta-Analysis. Ageing Res. Rev..

[293] Andersen S.L. (2020). Centenarians as Models of Resistance and Resilience to Alzheimer’s Disease and Related Dementias. Adv. Geriatr. Med. Res..

[294] Perls T.T. (2021). Cognitive Trajectories and Resilience in Centenarians—Findings from the 100-Plus Study. JAMA Netw. Open.

[295] Stern Y., Arenaza-Urquijo E.M., Bartrés-Faz D., Belleville S., Cantilon M., Chetelat G., Ewers M., Franzmeier N., Kempermann G., Kremen W.S. (2020). The Reserve, Resilience and Protective Factors PIA Empirical Definitions and Conceptual Frameworks Workgroup. Whitepaper: Defining and Investigating Cognitive Reserve, Brain Reserve, and Brain Maintenance. Alzheimers Dement..

[296] Arenaza-Urquijo E.M., Vemuri P. (2018). Resistance vs Resilience to Alzheimer Disease: Clarifying Terminology for Preclinical Studies. Neurology.

[297] Stern Y., Albert M., Barnes C.A., Cabeza R., Pascual-Leone A., Rapp P.R. (2023). A framework for concepts of reserve and resilience in aging. Neurobiol. Aging.

[298] Mortimer J.A., Borenstein A.R., Gosche K.M., Snowdon D.A. (2005). Very Early Detection of Alzheimer Neuropathology and the Role of Brain Reserve in Modifying Its Clinical Expression. J. Geriatr. Psychiatry Neurol..

[299] Stern Y. (2012). Cognitive Reserve in Ageing and Alzheimer’s Disease. Lancet Neurol..

[300] Arenaza-Urquijo E.M., Vemuri P. (2020). Improving the resistance and resilience framework for aging and dementia studies. Alzheimers Res. Ther..

